# Exploring the Distribution of the Spreading Lethal Salamander Chytrid Fungus in Its Invasive Range in Europe – A Macroecological Approach

**DOI:** 10.1371/journal.pone.0165682

**Published:** 2016-10-31

**Authors:** Stephan Feldmeier, Lukas Schefczyk, Norman Wagner, Günther Heinemann, Michael Veith, Stefan Lötters

**Affiliations:** 1 Department of Biogeography, Trier University, Trier, Germany; 2 Department of Environmental Meteorology, Trier University, Trier, Germany; National Zoological Park, UNITED STATES

## Abstract

The chytrid fungus *Batrachochytrium salamandrivorans* (*Bsal*) is a dangerous pathogen to salamanders and newts. Apparently native to Asia, it has recently been detected in Western Europe where it is expected to spread and to have dramatic effects on naïve hosts. Since 2010, *Bsal* has led to some catastrophic population declines of urodeles in the Netherlands and Belgium. More recently, it has been discovered in additional, more distant sites including sites in Germany. With the purpose to contribute to a better understanding of *Bsal*, we modelled its potential distribution in its invasive European range to gain insights about the factors driving this distribution. We computed *Bsal* Maxent models for two predictor sets, which represent different temporal resolutions, using three different background extents to account for different invasion stage scenarios. Beside ‘classical’ bioclimate, we employed weather data, which allowed us to emphasize predictors in accordance with the known pathogen’s biology. The most important predictors as well as spatial predictions varied between invasion scenarios and predictor sets. The most reasonable model was based on weather data and the scenario of a recent pathogen introduction. It identified temperature predictors, which represent optimal growing conditions and heat limiting conditions, as the most explaining drivers of the current distribution. This model also predicted large areas in the study region as suitable for *Bsal*. The other models predicted considerably less, but shared some areas which we interpreted as most likely high risk zones. Our results indicate that growth relevant temperatures measured under laboratory conditions might also be relevant on a macroecological scale, if predictors with a high temporal resolution and relevance are used. Additionally, the conditions in our study area support the possibility of a further *Bsal* spread, especially when considering that our models might tend to underestimate the potential distribution of *Bsal*.

## Introduction

Worldwide, biological diversity is alarmingly declining [1], and it has been proposed that we are witnessing a sixth mass extinction in Earth’s history [2]. Among the various reasons responsible for species and population declines and extinctions are emerging infectious diseases. There has been a remarkable pathogen-related increase in species declines during the last decades [3]. As pointed out by Daszak *et al*. (2000) [4], mediation by humans helps pathogens to cross evolutionary and ecological boundaries. Such a ‘pathogen pollution’ has led to dramatic species declines and extinctions in various regions [5,6].

Amphibians belong to the globally most threatened of all vertebrates. More than one third of the ca. 7,500 species is threatened with extinction when applying IUCN Red List criteria [1]. Emerging infectious diseases play a key role in the world’s ‘amphibian crisis’ and are seen as one main factor for the global amphibian decline [7]. The pandemic amphibian chytrid fungus (*Batrachochytrium dendrobatidis*, *Bd*) has significantly contributed to this crisis [8]. This pathogen is spreading and is already known from more than 400 host species in all three amphibian orders and on all continents where amphibians occur [8,9]. However, the effects of *Bd* infections depends on various factors, such as the *Bd* strain, the species, the population and regional climatic factors [8,9].

Recently, a congeneric chytrid fungus has been discovered in Europe [10]. *Batrachochytrium salamandrivorans* (*Bsal*) is lethal only to salamanders and newts, order Urodela. Under laboratory conditions, in many urodeles from outside Asia, *Bsal* infection led to 100% mortality within a few days [11]. Since this fungus has been found in wild Asian newts, it is expected that *Bsal* is native to Asia and has recently been introduced into Europe by humans [11]. Since 2010, observed Dutch populations of fire salamanders (*Salamandra salamandra*) have drastically declined due to *Bsal* infections, with sometimes less than one percent survival rate (the latter studied in one population only) [12,13]. In subsequent years, *Bsal* infections have been recorded in additional species and nearby sites (within a radius of about 100 km from first noted outbreaks) in Belgium and Germany, and it appears that *Bsal* is spreading in its invasive range [13]. According to these authors, to date *Bsal* infections in Europe in the wild are known in one salamander and two newt species at 14 sites. In addition, *Bsal* infections have recently been detected in captive salamanders and newts in the UK and Germany [14,15].

The emergence of *Bsal*, accompanied by massive amphibian diversity loss, is feared [11], especially in regions that are species-rich in salamanders and newts [16,17]. Biologists and conservationists are saying that *Bsal* mitigation strategies are necessary now. Recommendations for minimizing impacts of lethal emerging infectious diseases on wildlife, among others, include the reduction of local spread, habitat manipulation and host translocations [18]. Macroecological models forecasting the potential invasive range of pathogens are helpful in these steps [19]. Such ‘spatial risk assessments’ are available for the pandemic *Bd*, based on correlative species distribution models (SDMs). These use climatic information at pathogen presence records [20,21] to infer its environmental niche [22]. Until recently, the number of *Bsal* records was too few for SDM building. Aware of this circumstance, Yap *et al*. (2015) [23] provisionally used avatar species (i.e. records and random points inside the distribution of Asian urodeles that potentially can be *Bsal*-positive) to create a *Bsal* SDM for North America.

Besides using SDMs to predict distributions of species, they can be applied to gain insights about the environmental drivers of their distributions [24]. With this goal, we in this paper provide *Bsal* SDMs for the region where it is invasive in Europe. We use true observed presence records for SDM building. We create models with fine-scale weather data as well as ‘classical’ bioclimate data to explore the responses of *Bsal* with respect to different temporal resolutions, using predictors relevant to *Bsal* and adapted to known temperature limits [10]. A basic assumption of SDMs is that a species and its environment are in equilibrium [25,26]. SDMs built in early invasion stages, as it is expected for *Bsal* [13], are in conflict with this assumption and tend to underpredict the potential distribution of a species [27]. On the other hand, with regard to the development of mitigation strategies, attempting to build a *Bsal* SDM might be a valuable contribution. Our models were fine-tuned to better meet equilibrium conditions with respect to different invasion stages and according to the small number of presences to minimize biases. We consider our study as an important step towards a better understanding of *Bsal*.

## Materials and Methods

### Climate predictors and *Bsal* presence data

**Fig 1:**
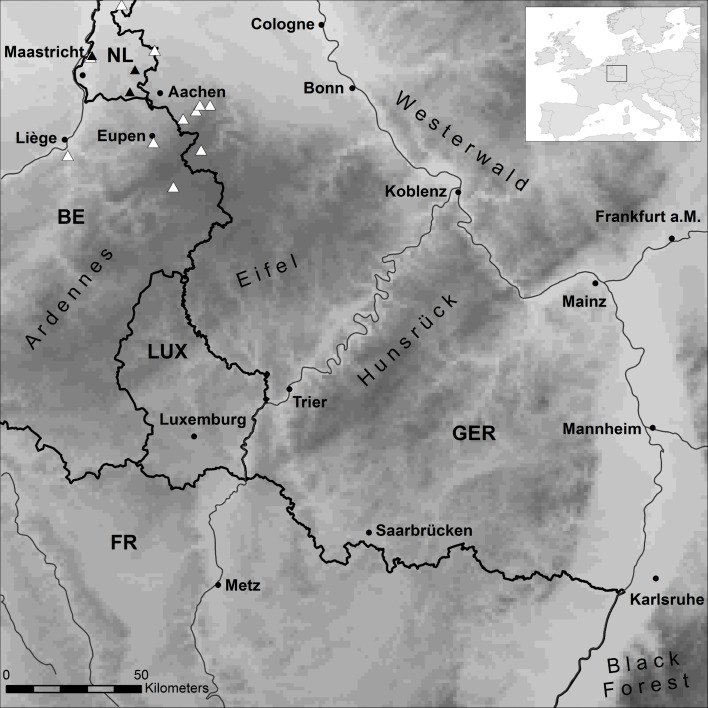
Western Europe study region. First noted *Bsal* outbreaks are indicated by black and subsequent records by white triangles. Elevational range (grey scales, light to dark: 28–1,050 m a.s.l.), borders (bold lines), rivers (thin lines), some major cities (normal font) and low mountain range names (sloped font) are shown.

The climate data used in this study were generated with the regional climate model COSMO-CLM (consortium for small-scale modelling—climate limited area modelling; [28]; http://www.clm-community.eu, version 5.0_clm6). It was forced with ERA-Interim data [29] and dynamically downscaled in a multi-nesting chain approach [30]. Hourly data of precipitation and surface temperature were modelled for the period from 1 January 2011 to 31 May 2014 at a horizontal resolution of ~ 1.3 km. We used the surface temperature because it is modelled with respect to seasonal vegetation cover, leaf area index, soil properties and others. As almost all *Bsal* records were located in forested areas, the surface temperature was supposed to reflect the conditions at ground level better than the standard 2 m air temperature. The climate data cover an area of 67,600 km^2^ (40,000 grid cells) in Belgium, France, Germany and the Netherlands (Fig 1). The area has lowlands (< 100 m a.s.l.), such as the Rhine valley and low mountain ranges with most of the highest peaks < 800 m a.s.l., including the Ardennes, Eifel, Westerwald, Hunsrück and parts of the Black Forest. The climate time period and the modelling domain was restricted due to limited computational resources and because it was set in the context of a different project (‘KlimLandRP’; http://www.klimlandrp.de). Despite these limitations, the application of this data is more appropriate than the use of standard Worldclim data [31], which strongly predates the *Bsal* presences and is not comparable in temporal resolution and quality.

Out of these data, we generated two initial predictor sets for our *Bsal* modelling approach: (1) We calculated 25 indices of weather extremes (S1 Table) of the ECA project (European Climate Assessment; http://www.eca.knmi.nl), which are based on hourly and daily surface temperatures and precipitation data over the three and a half year climate period (i.e. no yearly averages). To better meet the *Bsal* ecology, indices were adapted in a way that they acknowledged temperature growth limits of *Bsal* [10]: that is 5–25°C, with an optimum growth at 10–15°C. (2) Monthly multi-year averages of the precipitation as well as the minimum and maximum surface temperatures were taken to calculate 19 BIOCLIM variables [31,32] (S2 Table). BIOCLIM variables are biologically meaningful predictors and are widely used in SDM building [33]. These variables have a lower temporal resolution than the ECA indices. Hence, they do not or less account for short term extreme events, while ECA indices do.

Of the so far 14 *Bsal*-positive sites reported [10,11,13], 12 fell into the study region (Fig 1). The addition of two yet unpublished *Bsal*-positive sites allowed us to employ 14 records in the modelling process (S3 Table).

### Species distribution modelling

#### Model algorithm

The variety of algorithms available for correlative model computation can lead to markedly different SDM results. To account for this variation, it is generally recommended to employ distinct algorithms in SDM reconstructions [34–38]. On the other hand, Warton & Aarts (2013) [39] highlighted, that for distinct algorithms when applied in a similar way (i.e. the same random points, predictors, type of model responses), differences will typically be small and can even lead to almost identical results. However, steering a single algorithm in different ways can reveal dramatic output alterations [39]. Therefore, it is important to select a method, which is suitable for the available data and a modelling approach, which is ecologically reasonable [39,40]. In this study, we used Maxent 3.3.3k [41] to generate models. Maxent is a machine-learning algorithm, following the principle of maximum entropy. Thereby, it uses environmental predictors (here, out of the ECA and BIOCLIM sets), species presence records and random background points (at which the presence of the target species is unknown). It contrasts the environmental conditions at species’ presences against the conditions at the background points, to fit a function to estimate the relative habitat suitability [24,42,43].

We chose Maxent for our modelling approach for several reasons. We suggest that at the current state of knowledge a *Bsal* SDM should be based on presence-only information (i.e. applicable to Maxent). We reject the use of presence–absence, since a rigorous sampling for *Bsal* has just started in the invasive area [13]. For the same reason, we do not support the concept of pseudo-absence methods in this context. Besides, there is no clear strategy how to generate pseudo-absence and this is still subject of ongoing research [44–47]. Also, in correlative SDMs, model quality and accuracy depends on the number of records available of the target species. When the number of records is low (as in *Bsal*), Maxent has been shown to build reliable models and it can be fine-tuned to maximize the utility of the available information [48–52]. Maxent produces response curves, which allow to assess the modelled responses in terms of ecological meaning. Furthermore, via background definitions, it allows us to account for different dispersal hypotheses (i.e. different invasion stages) across the landscape. We acknowledge that in recent years Maxent has been scrutinized by some studies, especially because of its uncritical use in the literature [24,53,54]. However, due to the considerations above, we conclude that Maxent is the most appropriate method here, which we navigated with highest care.

#### Predictor selection

To avoid both model overfitting and data collinearity and because our goal was the interpretation of the environmental drivers of the distribution of *Bsal*, we reduced the number of predictors in our two predictor sets. This procedure leads to more parsimonious and interpretable models [24,55,56]. For this purpose, we examined pairwise Pearson correlation coefficients between the predictors and eliminated correlated variables with absolute values higher than 0.8 [57,58], because a more restrictive threshold value would have resulted in considerably less predictors. Moreover, we selected variables according to their expected relevance to *Bsal* life history [59]. The hypotheses behind this selection were based on *Bsal* in vitro temperature responses [10] as well as some general ecological aspects of the hosts [60]. Too cold or too hot conditions, likewise to dry conditions, should be disadvantageous to *Bsal*, as both fungal parasites and amphibian hosts generally rely on humid conditions. We choose predictors that reflected optimal conditions and limitations of *Bsal* [10], with emphasis of the adapted temperature indices (ECA set). Out of the ECA set, we therefore favoured predictors with a consecutive character over counting predictors. While, for instance, thirty consecutive days that are too hot for *Bsal* growth can be a limiting factor, thirty hot days might not be limiting when regularly interrupted by cooler days. However, in our study region these consecutive predictors are correlated to some counting predictors and can therefore be seen as surrogates for them (S4 Table).

Similar to the ECA approach, we chose a set out of the available BIOCLIM predictors that represent extreme and favourable conditions to *Bsal* (similar to [20,23]). The predictor ‘bio 8’ (mean temperature of wettest quarter) was included to account for possible temperature relationships at expected beneficial humid conditions, and ‘bio 15’ (precipitation seasonality) was included to examine the importance of stable precipitation conditions over the year. The resulting final predictor sets are shown in Table 1, S1 and S2 Figs, for correlations between final predictor sets and between the final and the initial predictor sets see S4 Table.

**Table 1:** Final predictor sets used for the *Bsal* SDMs.

Set	Code	Definition	Unit
ECA	csu 5	largest number of consecutive days where T_max_ > 5°C	days
	csu 25	largest number of consecutive days where T_max_ > 25°C	days
	su 10–15	number of days where 10°C < T_max_ < 15°C	days
	tr 10–15	number of days where 10°C < T_min_ < 15°C	days
	cddn	number of consecutive dry day (cdd) periods with ≥ 5 cdd per period	number
	cwdn	number of consecutive wet day (cwd) periods with ≥ 5 cwd per period	number
	r 10	number of days with precipitation < 10 mm	days
BIO	bio 8	mean temperature of wettest quarter	°C
	bio 10	mean temperature of warmest quarter	°C
	bio 11	mean temperature of coldest quarter	°C
	bio 15	precipitation seasonality (coefficient of variation)	-
	bio 16	precipitation of wettest quarter	mm
	bio 17	precipitation of driest quarter	mm

For additional information on ECA predictors, as explanations to codes, definitions of ‘dry’ or ‘wet’ days, see S1 Table.

#### Model fine-tuning and evaluation

We ran Maxent with the model complexity specifications described by Shcheglovitova & Anderson (2013) [51] for models with small sample sizes (only hinge features, regularization multiplier of 2). These authors found that models with more complex feature classes (which can lead to model overfitting) and with higher regularizations (which results in simpler models) outperform models with standard settings for small sample sizes (simple feature class with lower regularization). As Maxent assumes that a species is equally likely to be anywhere in the background extent, the latter is often restricted to areas that are accessible via dispersal, while modifying this extent is equivalent to changing the a priori expectation of a species’ distribution [24]. As it is presumed that *Bsal* is in an early invasion stage, regions in the south-western part of our study region may be far beyond the invasion front. As it is not clear if *Bsal* is really a recently introduced pathogen, as there are indications that *Bsal* has been introduced to Europe more than 10 years ago (authors’ unpubl. data), we defined three background extents to account for different hypothetical invasion scenarios (S3 Fig). We decided for an approach similar to Elith *et al*. (2010) [61] who compared a continent-wide background with a background restricted to a polygon in reachable distance around early invasion records. (1) As a conservative approach, we drew a minimum convex polygon (MCP) of 70 km distance (~ the known furthest distance of two *Bsal* records within the study region) around the presences to limit our background. This background represents the hypothesis of a recent arrival of *Bsal* in Europe and early invasive stage. (2) Additionally, we created a 150 km MCP (this distance accounts for the two presences outside our study region [13]) and (3) the full study area as alternative extents to account for the possibility of earlier unnoticed arrivals and a longer dispersal ability. Models, which were trained with a MCP background were again projected into the entire study area without ‘clamping’. When projecting a model into predictor conditions outside their training conditions, ‘no clamping’ results in continuing the response curve, while ‘clamping’ keeps the suitability constant at the limits of the training data [57]. We chose this setting because of the simple type of response curves. It seems reasonable to simply continue with them, as they are monotonic at their edges with no complex patterns. We present Multivariate Environmental Similarity Surfaces (MESS) maps [61] to identify areas outside of the training conditions of MCP-based models. In these areas, predictions could have poor support if the response curves are unrealistically extrapolated with respect to a species’ ecology [61].

Evaluating model performance for small sample sizes is a challenging task in SDM building for the lack of independent test data. To evaluate our models, we therefore employed a leave-one-out jackknife approach (in this case equal to a 14-fold cross-validation), which is applicable to small sample sizes [48,51]. Models were built for both predictor sets (ECA, BIO) and the three background extents (indexed ‘70’, ‘150’, ‘full’), respectively. We ran each model with 30 replicates to account for variance in the background data, using each time 10,000 different random background points. The area under the receiver operating characteristic curve (AUC) was calculated by Maxent as a measure of predictive accuracy, although we are aware that its use for presence-only data is controversially debated [54,62]. AUC values reflect the model’s ability to distinguish between presence and background points, giving information how general or restricted a distribution is along the range of the predictor variables in the study region [24,62]. AUC is considered useful to compare models built with the same data and background, which is not the case for the models in this study, as different background extents were used [24]. For this reason, we just state AUC as an indicator of how widespread the distribution of *Bsal* is predicted in our background area. Additionally, we present the omission of the test locality (test omission, proportion of misidentified test presences) with respect to the ‘minimum training presence’ (MTR) threshold, which is also calculated by Maxent [63]. For small sample sizes, this threshold rule represents a conservative approach, because it identifies the minimum predicted area, which keeps the omission error in the training data set at zero [48]. We also provide the predicted area for the binary maps with respect to the MTR threshold for each model type, calculated by Maxent. To identify the most important variables for each model, we used the analysis of variable contribution as incorporated in Maxent. We calculated for all measures the mean and standard deviation over all cross-validation folds and replicates. To compare the mean predictions of the different models we used Pearson correlation coefficients and Schoener’s D, a metric of niche overlap [64,65].

### Additional information on software and data used

We used CDO 1.6.2 [66] to process the climate model output (netCDF format) and to calculate the ECA indices. The R 3.2.2 [67] packages raster [68] and dismo [69] were employed for calculating BIOCLIM variables, raster correlations, niche overlap and boxplots. The pmcmr package [70] was used to apply the Kruskal-Wallis Nemenyi post-hoc test. ArcGIS 10.2.1 and SDMtoolbox 1.1b [71] were used to build the final predictor layers, check correlations between them and to create output maps and figures. Administrative boundary data was obtained from the GADM database 2.8 (http://www.gadm.org) and river data from the European Environment Agency, EEA (http://www.eea.europa.eu).

## Results

**Fig 2:**
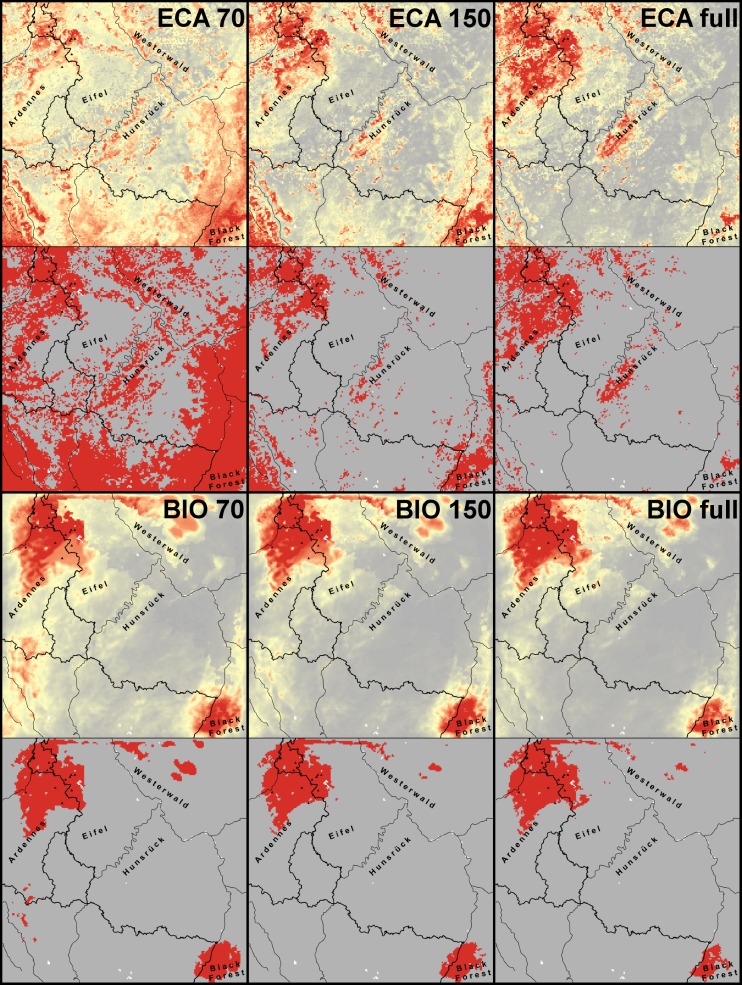
Predictions into the study region for the different *Bsal* SDMs. Predicted suitability maps (top) with corresponding presence-absence maps (below) for the different models. High suitability and presences are indicated in red, low suitability and absences in grey. *Bsal* presences are indicated by black triangles.

ECA_70_ performed best according to the test omission (Table 2). For both predictor sets the proportion of misidentified *Bsal* presence records increased with larger background extent, with the predicted area decreasing. Test AUC values mirrored the predicted area; less restricted predictions in the background extent (i.e. smaller AUC values) led to more widespread predictions over the entire study region. ECA_70_ predicted the largest and BIO_full_ the most restricted distribution of *Bsal* (Fig 2).

**Table 2:** Test omission, test AUC and predicted area for the different *Bsal* SDMs.

Model	Test omission	Test AUC	Predicted area
**ECA**_**70**_	0.079 ± 0.038 a	0.817 ± 0.004	0.436 ± 0.04 a
**ECA**_**150**_	0.290 ±0.082 b	0.896 ± 0.006	0.141 ± 0.01 bc
**ECA**_**full**_	0.271 ± 0.081 b	0.920 ± 0.006	0.125 ± 0.02 bd
**BIO**_**70**_	0.143 ± 0.000 c	0.871 ± 0.001	0.275 ± 0.00 ac
**BIO**_**150**_	0.143 ± 0.000 c	0.941 ± 0.002	0.114 ± 0.00 de
**BIO**_**full**_	0.214 ± 0.000 b	0.952 ± 0.002	0.084 ± 0.00 e

Values are mean ± one SD. Test omission and predicted area were calculated with respect to the ‘minimum training presence’ threshold. Value pairs with no letter in common are significantly different (Kruskal-Wallis test, Nemenyi post-hoc, p < 0.05). As AUC values are not comparable between models, no statistical test was applied.

The BIO models were almost identical to each other, independent from the background selection, which was reflected by high values of Pearson correlation coefficient and Schoener’s D (Table 3). Suitable areas were mainly predicted in the region of the known *Bsal* presences, into small parts of the Westerwald and the northern portion of the Black Forest. On the contrary, ECA models differed more strongly from each other, most obvious between ECA_70_ and ECA_full_ (Fig 2, Table 3). ECA_70_ predicted suitable areas all over the study area, with larger areas of unsuitability only in the Eifel, Westerwald and area between the Hunsrück and the Black Forest. The other ECA models predicted considerably less suitable areas to *Bsal*. They identified similar suitable regions as did the BIO models, with some additional areas in the Hunsrück and the south-western portion of the study region.

**Table 3:** Pairwise Pearson correlation coefficients r and Schoener’s D (grey-shaded) for average ECA and BIO model predictions.

r \ D	ECA_70_	ECA_150_	ECA_full_	r \ D	BIO_70_	BIO_150_	BIO_full_
**ECA**_**70**_	1	0.795	0.701	**BIO**_**70**_	1	0.840	0.799
**ECA**_**150**_	0.836	1	0.797	**BIO**_**150**_	0.960	1	0.921
**ECA**_**full**_	0.451	0.784	1	**BIO**_**full**_	0.929	0.982	1

For ECA_70_ and ECA_150_, the temperature-based predictors ‘tr 10–15’ (days with minimum temperatures between 10° and 15°C) and ‘csu 25’ (highest number of consecutive days warmer than 25°C) were the most contributing variables. The other predictors contributed noticeably less (Table 4). In ECA_full_ though, ‘tr 10–15’ lost importance and ‘csu 25’ became the most explaining variable, followed by ‘cddn’ (number of dry periods) and ‘csu 5’ (highest number of consecutive days warmer than 5°C). Among all BIO models, ‘bio 11’ and ‘bio 17’ clearly were the most important variables. Associated variable response curves are provided in S4 Fig, and MESS maps to assess response extrapolation in S5 Fig.

**Table 4:** Relative variable contributions of the predictor variables to *Bsal* SDMs.

	**ECA**_**70**_		**ECA**_**150**_		**ECA**_**full**_	
	**pc**	**pi**	**pc**	**pi**	**pc**	**pi**
**csu 25**	**34.4**	**32.8**	**26.5**	**24.0**	**39.4**	**49.4**
**csu 5**	0.0	0.0	6.4	4.1	**18.8**	**20.8**
**su 10–15**	0.0	0.0	5.9	0.3	6.5	1.7
**tr 10–15**	**56.2**	**55.8**	**32.1**	**48.0**	4.0	10.9
**cddn**	0.3	0.5	**19.5**	7.1	**30.1**	**15.2**
**cwdn**	0.8	1.4	2.7	4.6	0.5	0.7
**r 10**	**8.3**	**9.5**	7.0	**11.8**	0.7	1.3
	**BIO**_**70**_		**BIO**_**150**_		**BIO**_**full**_	
	**pc**	**pi**	**pc**	**pi**	**pc**	**pi**
**bio 8**	**21.6**	**8.4**	9.2	**6.0**	8.1	**7.0**
**bio 10**	0.0	0.0	0.0	0.0	2.1	4.1
**bio 11**	**35.8**	**50.2**	**37.3**	**46.3**	**26.7**	**43.8**
**bio 15**	3.0	1.1	**12.6**	2.6	**21.1**	3.3
**bio 16**	0.0	0.0	0.0	0.0	0.0	0.0
**bio 17**	**39.6**	**40.2**	**40.9**	**45.0**	**42.0**	**41.9**

Variable contributions are shown as average percent contribution (pc) and permutation importance (pi) (for details see https://www.cs.princeton.edu/~schapire/maxent/tutorial/tutorial.doc). For each model the three highest values are in bold.

## Discussion

### Model performance

Since SDMs infer the environmental niche of a species from its presences as well as from environmental data [22], it can be problematic if the number of presence data is too small to cover all suitable conditions to describe a species’ niche. Because of the small number of known *Bsal* presences and the expected Asian origin of this pathogen [11], it has to be taken into account that our models do not reflect the entire niche of *Bsal*. In addition, it should be considered that occasionally niche shifts occur during biological invasions [72,73]. As a consequence, our *Bsal* SDMs more represent ‘conservative’ approaches, which likely underestimate the potential distribution of *Bsal* [27]. Thus, they emphasize the predicted suitable areas. Nevertheless, as our main goal was to shed some light into the driving factors of *Bsal* distribution, our modelling approach is appropriate.

Among our models, ECA_70_ performed best. As ECA predictors were customised according to *Bsal* temperature physiology and as the restricted background presumably reflects a more realistic invasion scenario, we suggest this to be a reasonable result. However, some uncertainty is casted on this, because of the second best performing models, BIO_70_ and BIO_150_. They markedly differed in their predictions in geographic space, while performing only slightly less well with regard to test omission. Differences between the various background extents, especially with respect to the predicted area, are not surprising. As a larger background usually goes hand in hand with a wider range of predictor values, it is easier for a model to fit responses to contrast the presences against the background (compare predictor responses for the different backgrounds, S4 Fig). As a consequence, the predicted *Bsal* distribution is more restricted around the presences. In reverse, projections from smaller backgrounds into the full area are less restricted.

Since almost all *Bsal* presences are located in forested areas and as the known amphibian host species in our study area are ground-dwelling, we accentuated the surface temperature for the calculation of the temperature variables. Through the incorporation of the effect of vegetation cover, we expected that our temperature-based predictors well reflect the niche of *Bsal*. Additionally, the shapes of all response curves were reasonable and supported the hypotheses, which were considered during the process of variable selection. The response of ‘bio 8’ appears to be uncommon, but is sound when accounting for the fact that the wettest quarter is associated to cold temperatures in the study region. Therefore, too warm conditions in winter might correlate with too hot conditions in summer. As the predictor ranges in the full study region compared to the restricted background extent were pretty similar (see MESS maps, S5 Fig [61]), questionable extrapolations into new predictor conditions were not problematic.

### Predictor importance

The temporal more coarse BIO models were mainly driven by ‘bio 11’ (mean temperature of coldest quarter) and ‘bio 17’ (precipitation of driest quarter), representing an approach that identifies regions according to the modelled lower limits of temperature and precipitation suitable to *Bsal*. These findings are in concordance with the known needs of *Bsal* under laboratory conditions, but depicted a rather simple model, as the expected upper temperature limit [10] was not found or reached by the mean monthly temperature. As hosts often die from *Bsal* within a few days after infection [11], short time weather events might be more relevant to *Bsal* survival and added valuable extra information to our models. Several studies have brought to light that SDMs benefit from the incorporation of weather extremes [74–77]. This was acknowledged by the ECA predictor set. ECA models with restricted background were mainly influenced by ‘tr 10–15’, i.e. the number of days where the minimum temperature lays in the optimal growth temperature range of *Bsal*. Opposed to the BIO models, the second most important variable was also temperature-related, namely ‘csu 25’, the maximum length of heat periods. Such temperature-based predictors seem plausible to explain the current distribution of *Bsal*, because in previous laboratory experiments colonization of hosts occurred at temperatures above 15°C, but the critical thermal maximum for *Bsal* was 25°C [10,78,79]. A precipitation-based predictor, ‘cddn’ (number of dry periods), became again more influent for ECA_full_. These findings indicate that temperature conditions may play a more important role to *Bsal* than precipitation, at least in our study region. Furthermore, high temperatures might be a stern limiting factor to the fungus, even on a landscape scale. However, to identify these limiting temperatures, predictor variables may be needed with a high temporal resolution, rather than data based on monthly averages.

### Assessing *Bsal* high risk zones

So far, *Bsal* is known from a limited number of records only, but is expected to spread [13]. The known host species in the pathogen’s invasive European range are, when put together, distributed more or less evenly throughout our study region [80]. This implies that unlimited pathogen spread is a possible scenario, even when amphibians are the only *Bsal* vectors. Even worse, if additionally other spread pathways play a role, such as human-mediated long distance dispersal (as known in *Bd* [8]). For these reasons, an essential question is, where in our study region *Bsal* is able to cope with the abiotic environment. Even when we are unable to fully answer this question with the present SDMs, because of the fungus’ cryptic niche as a result of its expected non-equilibrium stage, these SDMs help to gain knowledge on high risk zones of *Bsal* invasion. In conservation practice, this is an important first step when developing mitigation strategies [18] and justifies a *Bsal* SDM approach despite its presumed early invasion stage.

**Fig 3:**
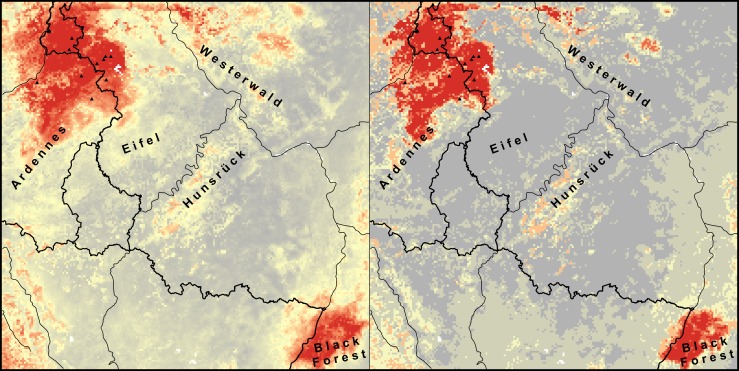
Average predictions of all *Bsal* SDMs. Average predicted suitability map (left) and presence-absence map (right) of all models. High values are indicated in red, low values in grey. *Bsal* presences are indicated by black triangles.

Certain areas within the study region were identified as highly suitable to *Bsal* by all modelling approaches (Fig 3). This included the north-western portion, i.e. parts of the Ardennes and the Eifel; likewise, parts of the Westerwald and the Black Forest. We suggest these to represent ‘most likely high risk zones’. All ECA models additionally supported high suitability in the Hunsrück. Moreover, these models suggested patchily distributed high suitability in-between the areas mentioned. In ECA_70_, which was considered as the best model (Table 2), this resulted in the largest extant of high suitability covering half of our study region (Fig 2), supporting the future scenario of further spread of the pathogen. As the ECA approach accounted for weather extremes, which might be informative in the case of *Bsal* (see above), we propose these areas to represent ‘likely high risk zones’.

## Conclusion

We identified the apparently most important predictors for the present distribution of the fungus for the lethal amphibian pathogen *Batrachochytrium salamandrivorans* in its invasive range in Europe. Different SDMs with both bioclimate and fine-scale weather data helped us to further identify possible risk zones. The most important predictors of the best fitting model were temperature-based (number of days with minimum temperature > 10°C and < 15°C and the largest number of consecutive days where the maximum temperature was > 25°C). This seems plausible with regard to the (still limited) information on the biology of this recently discovered pathogen and indicates that growth limiting temperature values of laboratory experiments are also relevant on a landscape scale. The best model predicted suitable areas all over the study area, with larger areas of unsuitability only in parts of the Eifel, Westerwald and the area between the Hunsrück and the Black Forest, indicating that a further *Bsal* spread is a possible scenario. Especially because it is known that niche shifts can occur during biological invasions and as we probably did not capture the entire niche of *Bsal*, our models may underpredict the potential distribution of this amphibian pathogen in Europe. Nevertheless, as our main goal was to shed some light into the driving factors of *Bsal* distribution, our modelling approach was reasonable and can be helpful for further *Bsal* exploration and SDMs.

## Supporting Information

S1 Fig Maps of the ECA predictor set. (PDF)

S2 Fig Maps of the BIO predictor set. (PDF)

S3 Fig Background restricting minimum convex polygons (MCPs). (PDF)

S4 Fig Model response curves. (PDF)

S5 Fig MESS maps. (PDF)

S1 Table ECA predictor variables. (DOCX)

S2 Table BIOCLIM predictor variables. (DOCX)

S3 Table *Bsal* presences. (DOCX)

S4 Table Predictor correlations. (DOCX)

S1 ZIP Archive ECA predictors (initial set, full extent). (ZIP)

S2 ZIP Archive BIOCLIM predictors (initial set, full extent). (ZIP)
