# Supplementary figures and images for: Exploring the Distribution of the Spreading Lethal Salamander Chytrid Fungus in Its Invasive Range in Europe – A Macroecological Approach
Source: PLoS One. 2016 Oct 31;11(10):e0165682. doi: 10.1371/journal.pone.0165682 (PMC5087956; doi:10.1371/journal.pone.0165682)

**cddn**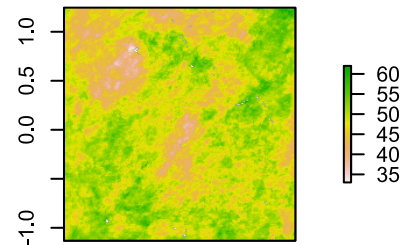**csu25**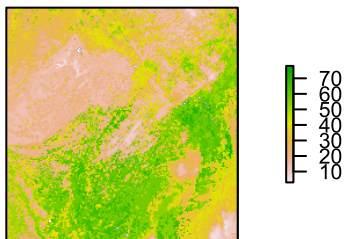**csu5**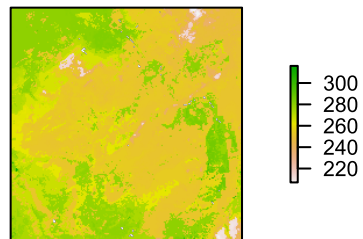**cwdn**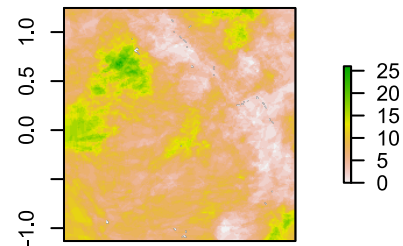**r10**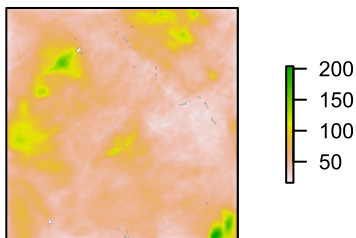**su10\_15**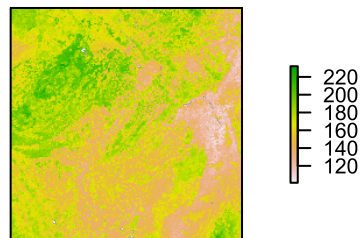**tr10\_15**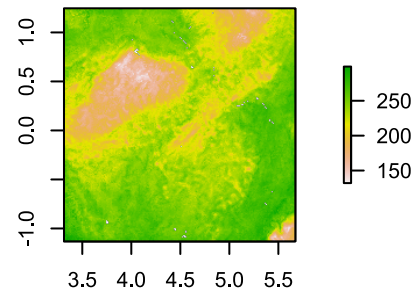

Supplement: S1 Fig — (PDF) [file pone.0165682.s001.pdf]

**bio 10**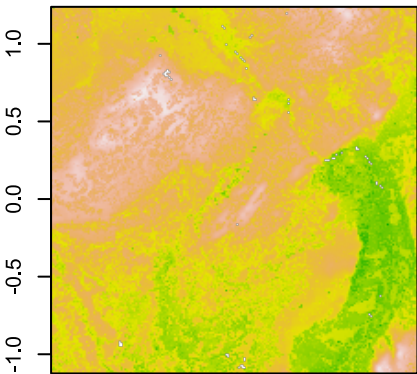**bio 11**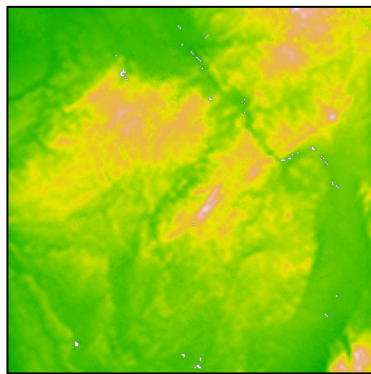**bio 15**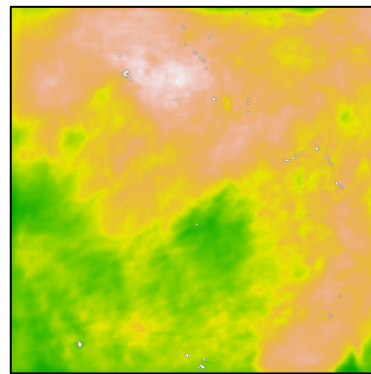**bio 16**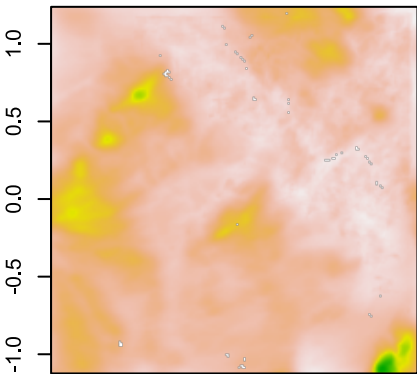**bio 17**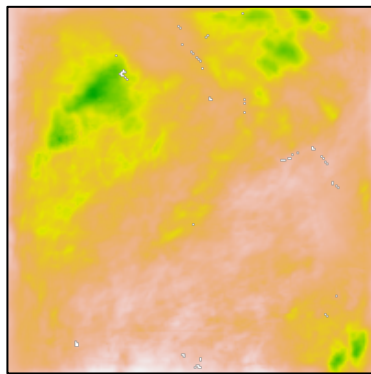**bio 8**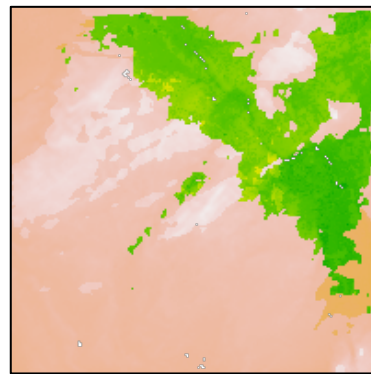

Supplement: S2 Fig — (PDF) [file pone.0165682.s002.pdf]
