# Supplementary material for: Exploring the Distribution of the Spreading Lethal Salamander Chytrid Fungus in Its Invasive Range in Europe – A Macroecological Approach
Source: PLoS One. 2016 Oct 31;11(10):e0165682. doi: 10.1371/journal.pone.0165682 (PMC5087956; doi:10.1371/journal.pone.0165682)
Supplement: S3 Fig — (PDF) [file pone.0165682.s003.pdf]

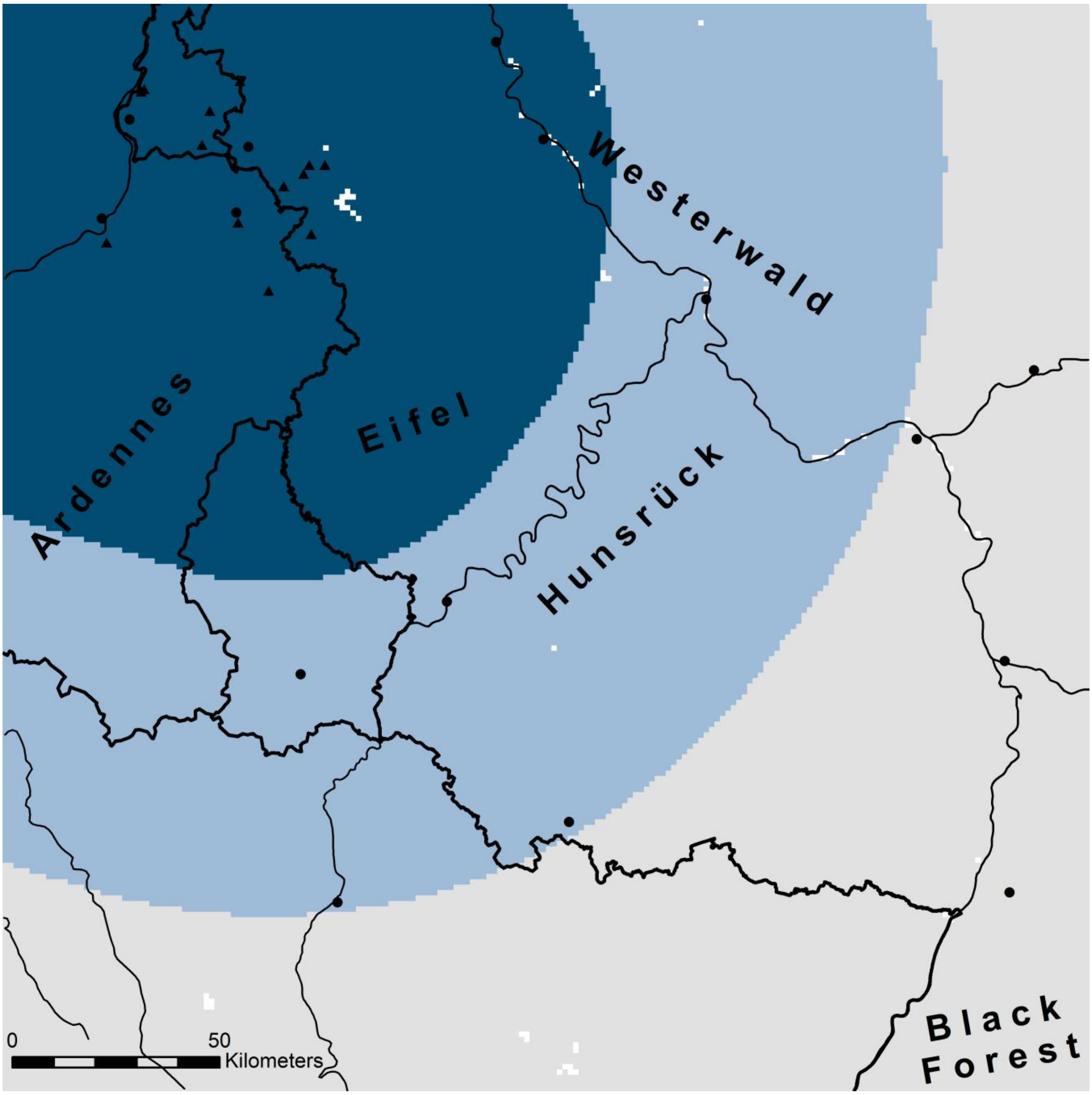

S3 Fig. Background restricting minimum convex polygons (MCPs).

MCP 70 km: dark blue area; MCP 150 km: dark and light blue area.
