# Supplementary material for: Exploring the Distribution of the Spreading Lethal Salamander Chytrid Fungus in Its Invasive Range in Europe – A Macroecological Approach
Source: PLoS One. 2016 Oct 31;11(10):e0165682. doi: 10.1371/journal.pone.0165682 (PMC5087956; doi:10.1371/journal.pone.0165682)
Supplement: S4 Fig — (PDF) [file pone.0165682.s004.pdf]

S4 Figures

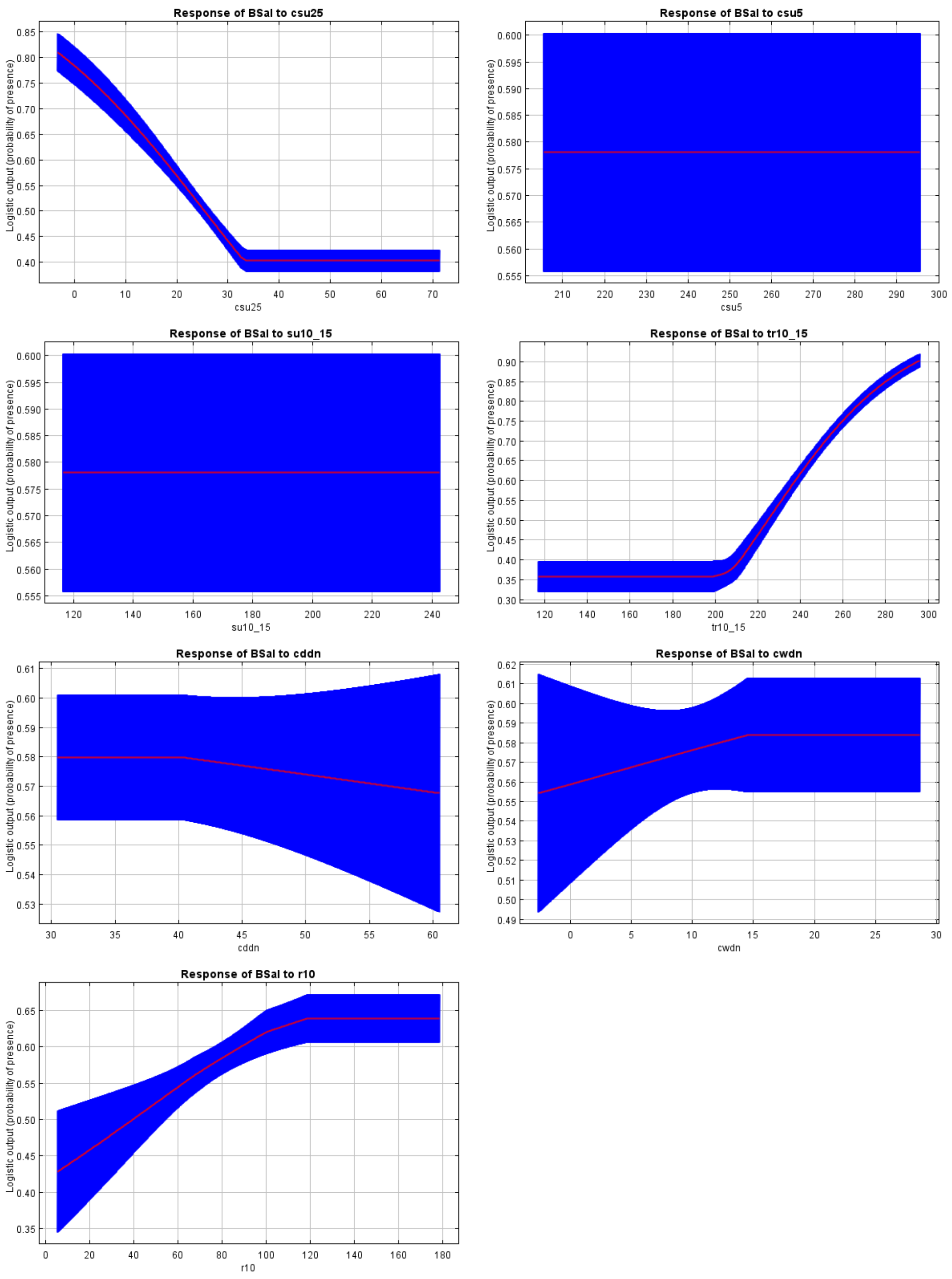

S2 Fig A. ECA$_{70}$ response curves.

Exemplary mean responses of the 14 cross-validation runs ± one standard deviation for one replicate run of the ECA$_{70}$ model. The curves show the logistic output as a function of each variable, while all other variables are kept at their average value. Note different scales on the y-axis.

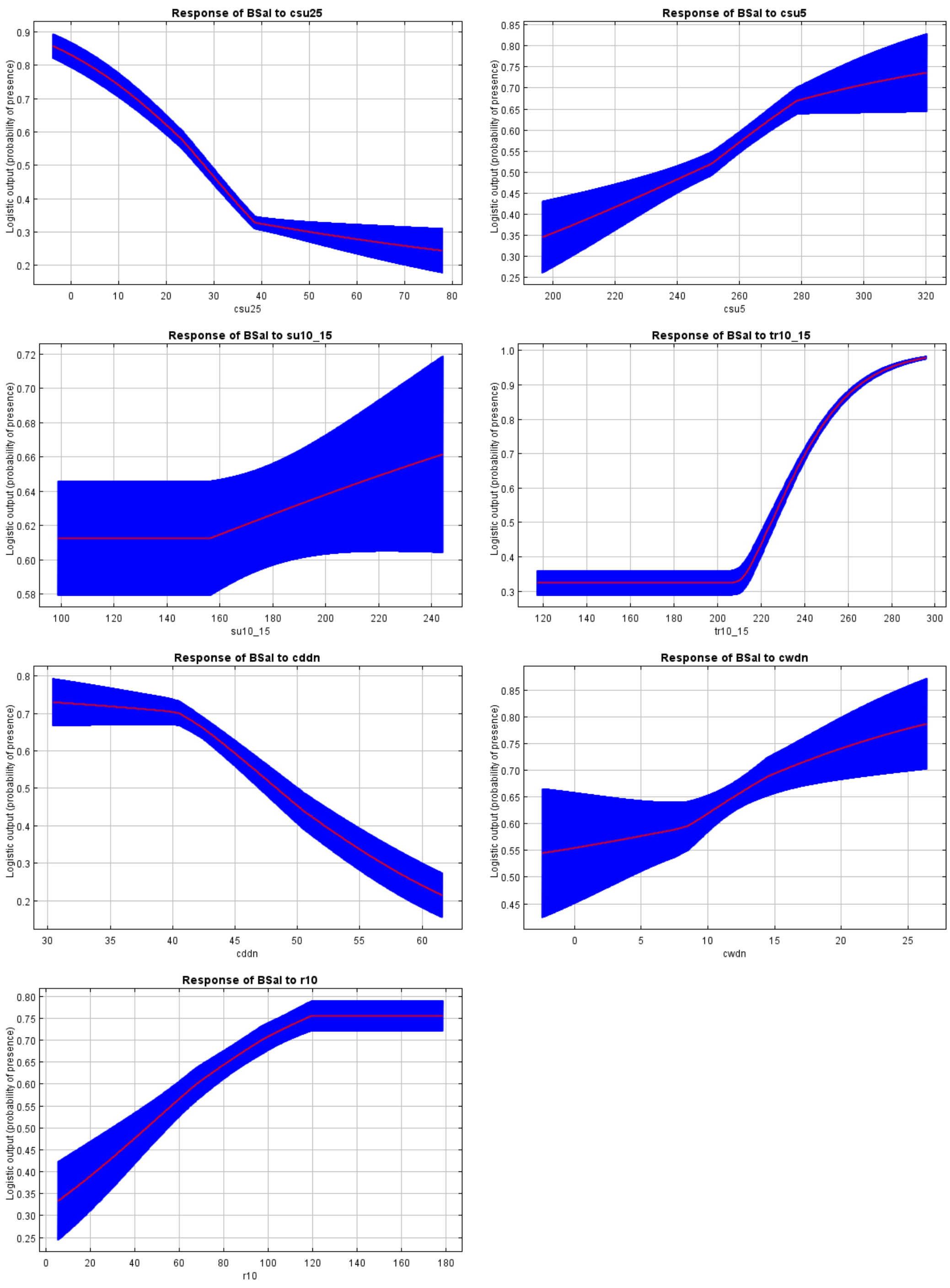

S2 Fig B. ECA_{150} response curves.

Exemplary mean responses of the 14 cross-validation runs ± one standard deviation for one replicate run of the ECA_{150} model. The curves show the logistic output as a function of each variable, while all other variables are kept at their average value. Note different scales on the y-axis.

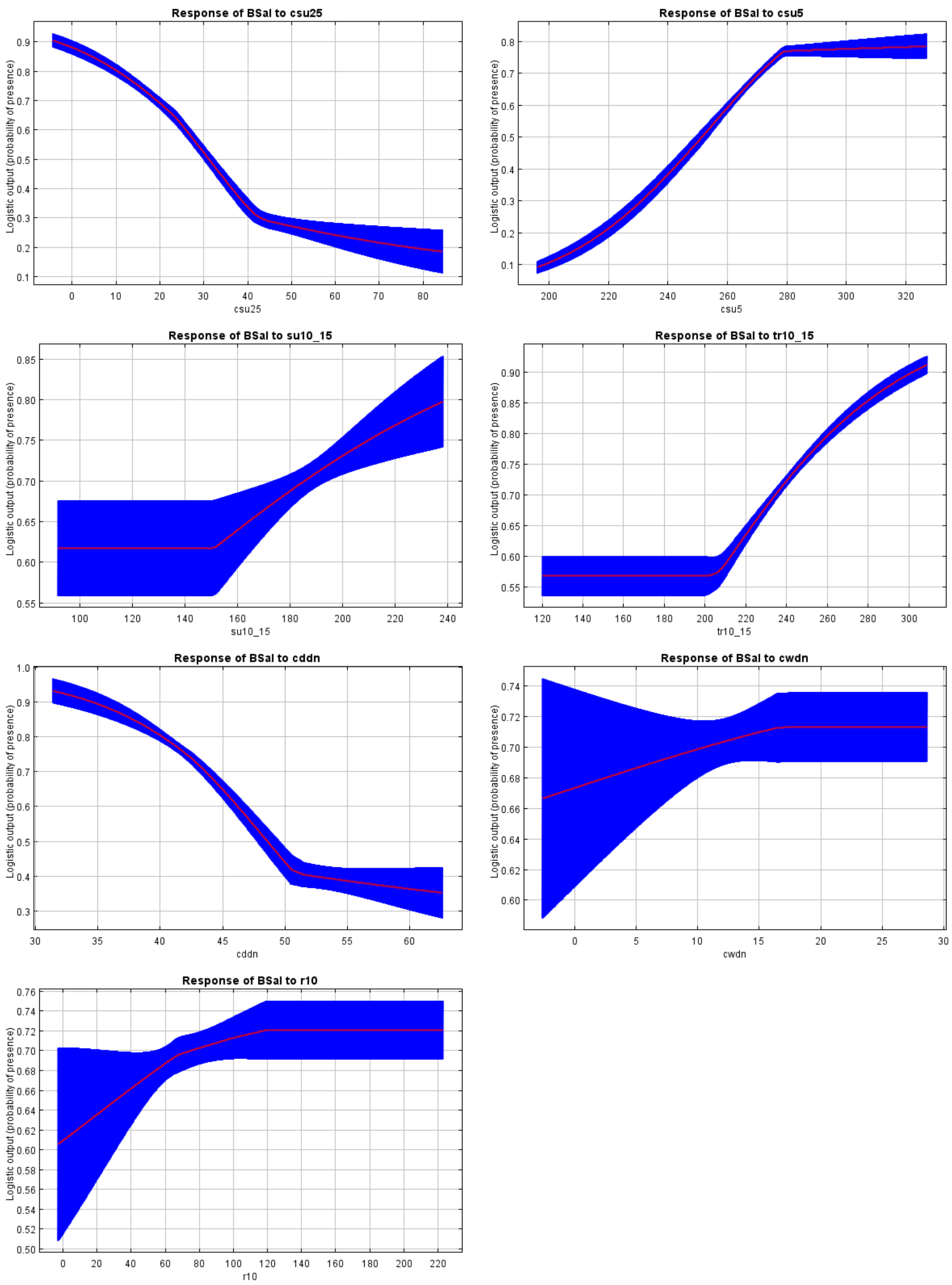

S2 Fig C. ECA$_{full}$ response curves.

Exemplary mean responses of the 14 cross-validation runs ± one standard deviation for one replicate run of the ECA$_{full}$ model. The curves show the logistic output as a function of each variable, while all other variables are kept at their average value. Note different scales on the y-axis.

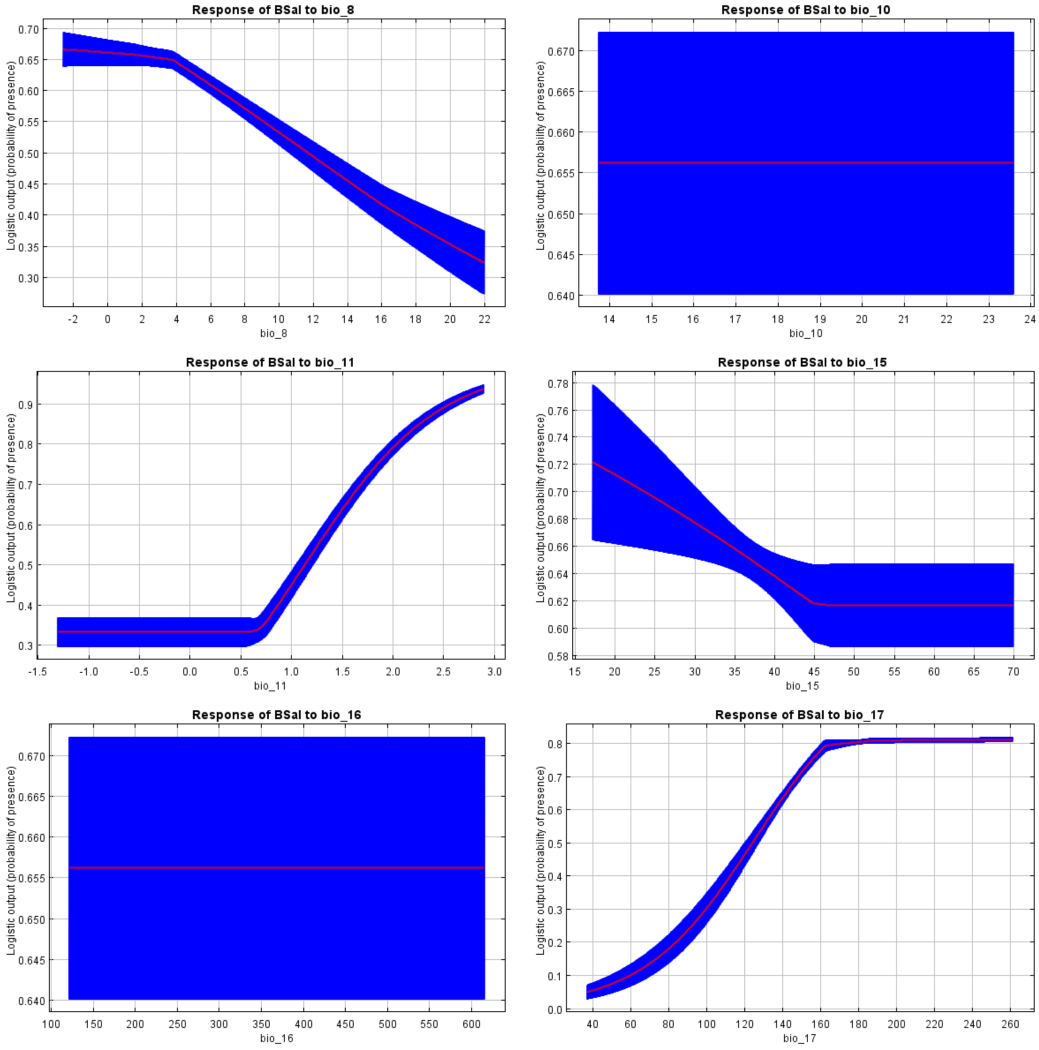

S2 Fig D. BIO_{70} response curves.

Exemplary mean responses of the 14 cross-validation runs ± one standard deviation for one replicate run of the BIO_{70} model. The curves show the logistic output as a function of each variable, while all other variables are kept at their average value. Note different scales on the y-axis.

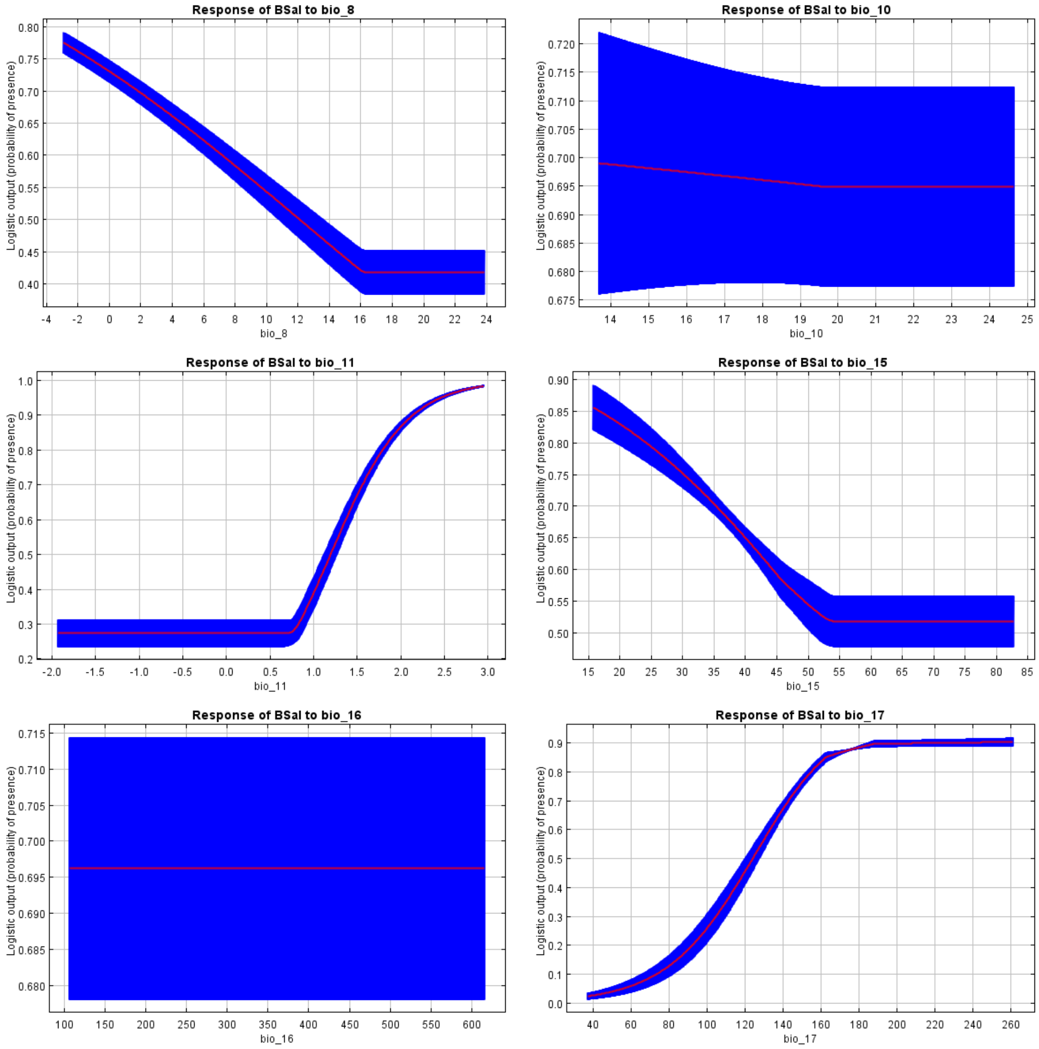

S2 Fig E. BIO_{150} response curves.

Exemplary mean responses of the 14 cross-validation runs ± one standard deviation for one replicate run of the BIO_{150} model. The curves show the logistic output as a function of each variable, while all other variables are kept at their average value. Note different scales on the y-axis.

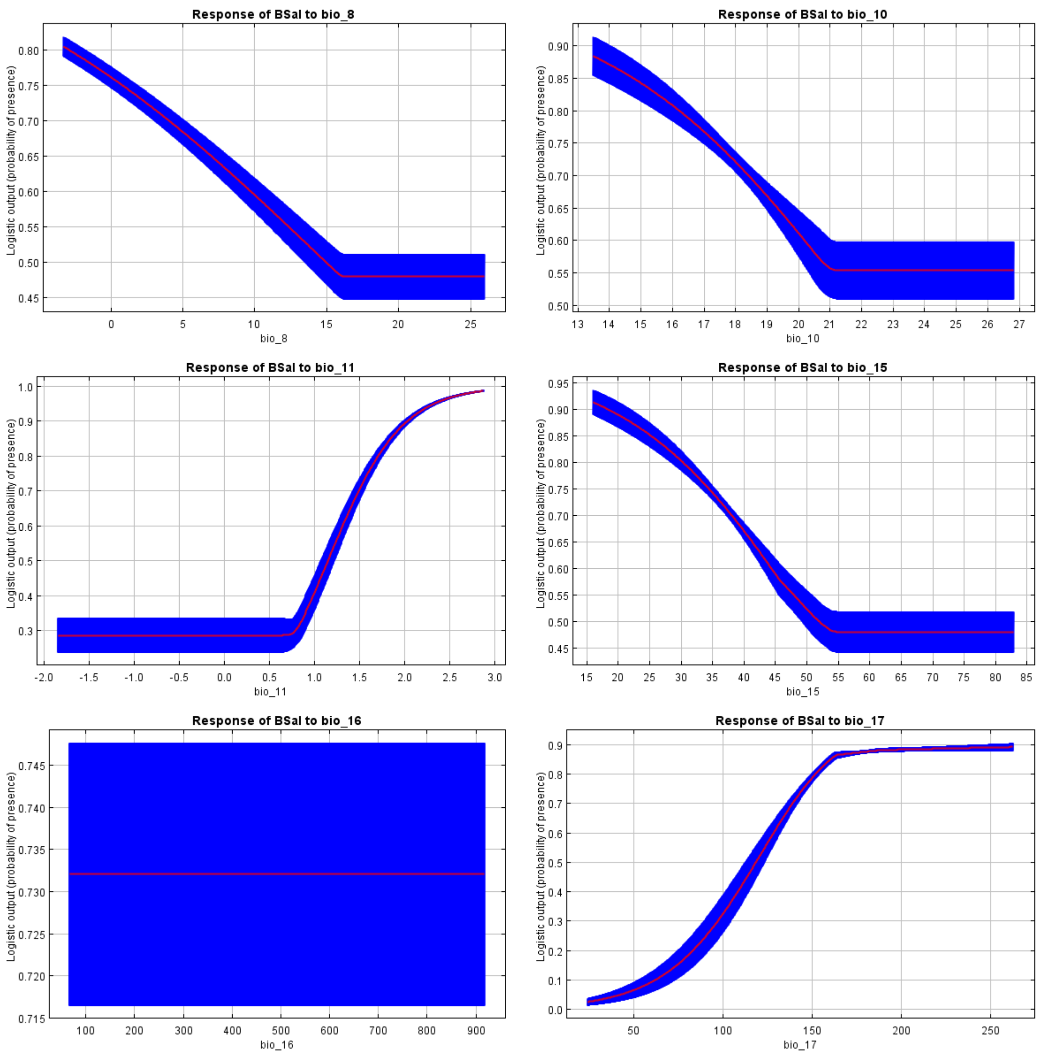

S2 Fig F. BIO_{full} response curves.

Exemplary mean responses of the 14 cross-validation runs ± one standard deviation for one replicate run of the BIO_{full} model. The curves show the logistic output as a function of each variable, while all other variables are kept at their average value. Note different scales on the y-axis.
