# Supplementary material for: Exploring the Distribution of the Spreading Lethal Salamander Chytrid Fungus in Its Invasive Range in Europe – A Macroecological Approach
Source: PLoS One. 2016 Oct 31;11(10):e0165682. doi: 10.1371/journal.pone.0165682 (PMC5087956; doi:10.1371/journal.pone.0165682)
Supplement: S5 Fig — (PDF) [file pone.0165682.s005.pdf]

S5 Figures

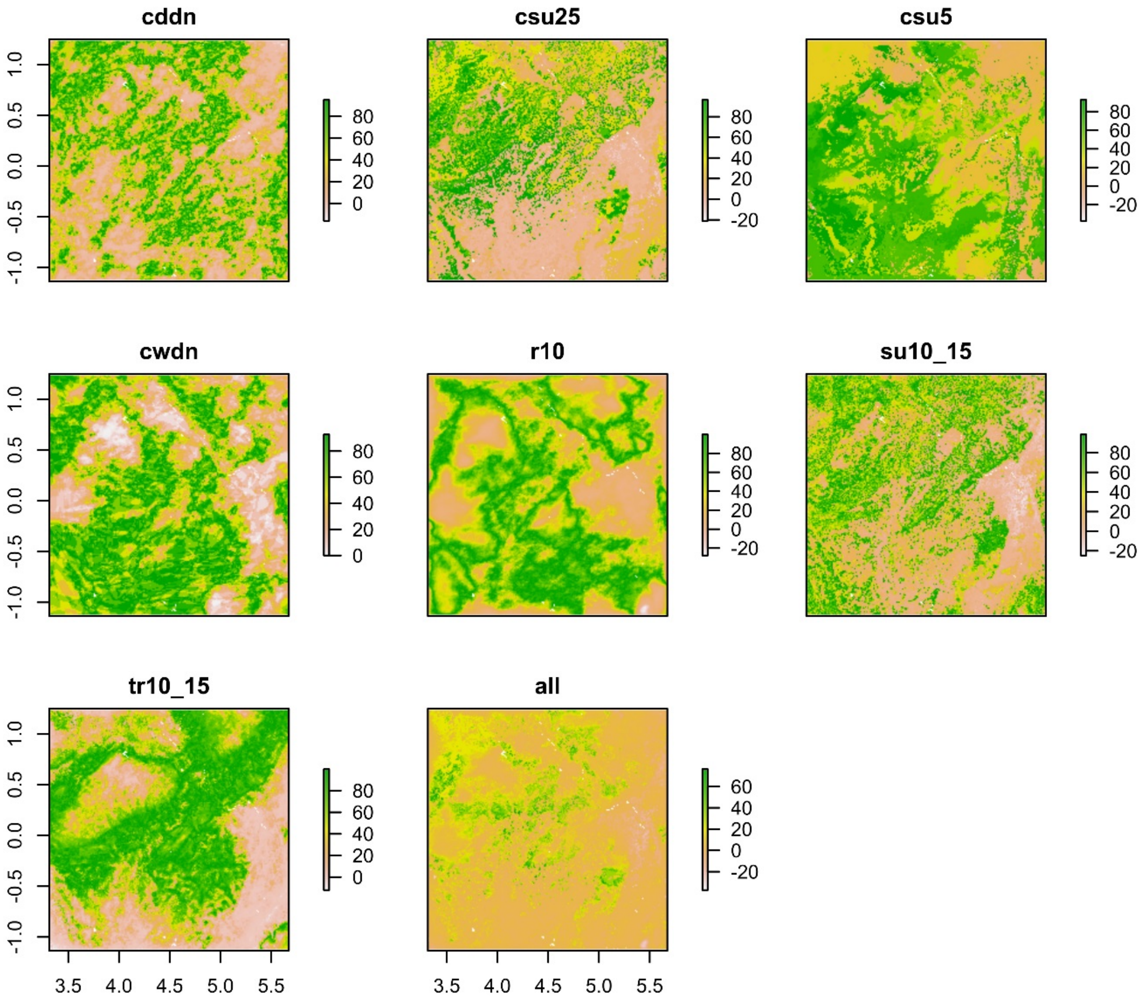

S3 Fig A. ECA MCP 70 MESS maps.

Multivariate environmental similarity surface (MESS) maps for the ECA predictor set. Reference are the conditions of the ECA predictor set at the MCP 70 background. Negative values indicate conditions outside their training range.

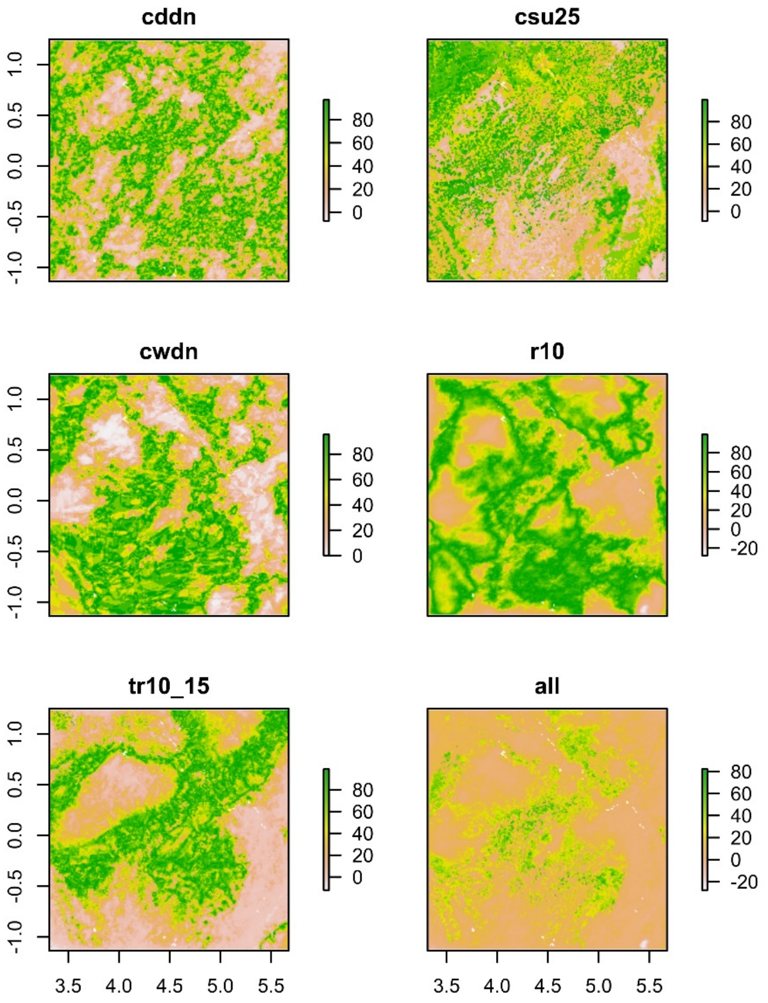

S3 Fig B. ECA MCP 150 MESS maps.

Multivariate environmental similarity surface (MESS) maps for the ECA predictor set. Reference are the conditions of the ECA predictor set at the MCP 150 background. Negative values indicate conditions outside their training range.

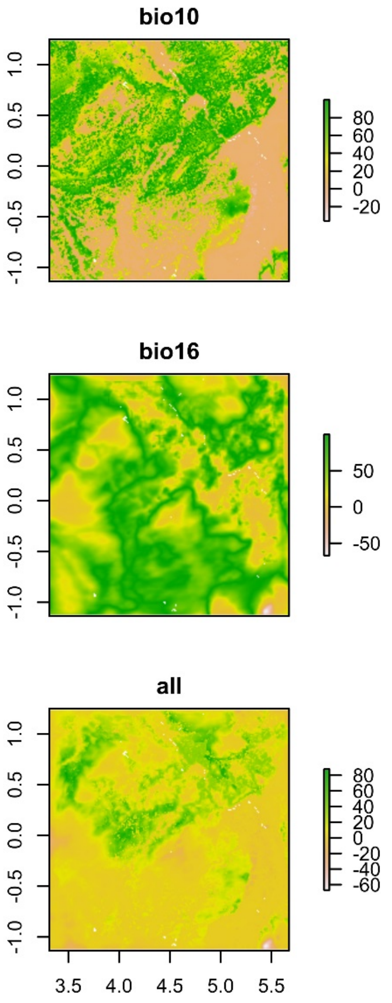

S3 Fig C. BIO MCP 70 MESS maps.

Multivariate environmental similarity surface (MESS) maps for the BIO predictor set. Reference are the conditions of the BIO predictor set at the MCP 70 background. Negative values indicate conditions outside their training range.

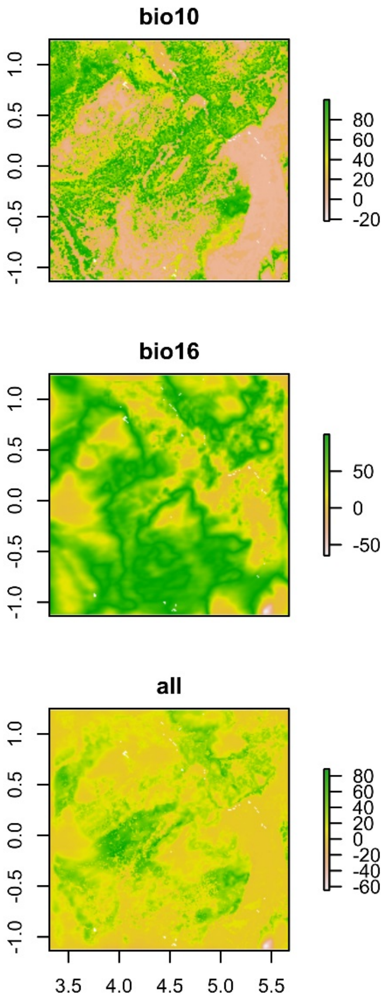

S3 Fig D. BIO MCP 150 MESS maps.

Multivariate environmental similarity surface (MESS) maps for the BIO predictor set. Reference are the conditions of the BIO predictor set at the MCP 150 background. Negative values indicate conditions outside their training range.
