# Supplementary material for: Exploring the Distribution of the Spreading Lethal Salamander Chytrid Fungus in Its Invasive Range in Europe – A Macroecological Approach
Source: PLoS One. 2016 Oct 31;11(10):e0165682. doi: 10.1371/journal.pone.0165682 (PMC5087956; doi:10.1371/journal.pone.0165682)
Supplement: S1 Table — (DOCX) [file pone.0165682.s006.docx]

**S1 Table. ECA predictor variables.**

| **Code** | **Description** | **Definition** | **Unit** |
| --- | --- | --- | --- |
| cdd | consecutive dry days | max. number of consecutive days with precipitation < 1 mm | days |
| cddn | dry periods | number of cdd periods with ≥ 5 cdd per period | number |
| cwd | consecutive wet days | max. number of cwd with precipitation > 1 mm | days |
| cwdn | wet periods | number of cwd periods with ≥ 5 cwd per period | number |
| r 1/10/20 | precipitation days | number of days with precipitation < 1/10/20 mm | days |
| su 5/10/15/20/25 | summer days | number of days where T_max_ > 5/10/15/20/25 °C | days |
| su 10-15 | summer days | number of days where 10 °C < T_max_ < 15 °C | days |
| csu 5/10/15/20/25 | consecutive summer days | max. number of consecutive summer days where T_max_ > 5/10/15/20/25 °C | days |
| tr 10/15/20 | tropical nights | number of days where T_min_ > 10/15/20 °C | days |
| tr 10-15 | tropical nights | number of days where 10 °C < T_min_ < 15 °C | days |
| fd | frost days | number of days where T_min_ < 0 °C | days |
| cfd | consecutive frost days | max. number of consecutive frost days | days |
| id | ice days | number of days where T_max_ < 0 °C | days |
