# Supplementary material for: Exploring the Distribution of the Spreading Lethal Salamander Chytrid Fungus in Its Invasive Range in Europe – A Macroecological Approach
Source: PLoS One. 2016 Oct 31;11(10):e0165682. doi: 10.1371/journal.pone.0165682 (PMC5087956; doi:10.1371/journal.pone.0165682)
Supplement: S2 Table — (DOCX) [file pone.0165682.s007.docx]

**S2 Table. BIOCLIM predictor variables.**

| **Code** | **Description** | **Unit** |
| --- | --- | --- |
| bio 1 | Annual Mean Temperature | °C |
| bio 2 | Mean Diurnal Range (Mean of monthly (max temp - min temp)) | °C |
| bio 3 | Isothermality (bio 2 / bio 7) (* 100) | % |
| bio 4 | Temperature Seasonality (standard deviation *100) | °C *100 |
| bio 5 | Max Temperature of Warmest Month | °C |
| bio 6 | Min Temperature of Coldest Month | °C |
| bio 7 | Temperature Annual Range (bio 5 – bio 6) | °C |
| bio 8 | Mean Temperature of Wettest Quarter | °C |
| bio 9 | Mean Temperature of Driest Quarter | °C |
| bio 10 | Mean Temperature of Warmest Quarter | °C |
| bio 11 | Mean Temperature of Coldest Quarter | °C |
| bio 12 | Annual Precipitation | mm |
| bio 13 | Precipitation of Wettest Month | mm |
| bio 14 | Precipitation of Driest Month | mm |
| bio 15 | Precipitation Seasonality (Coefficient of Variation) | - |
| bio 16 | Precipitation of Wettest Quarter | mm |
| bio 17 | Precipitation of Driest Quarter | mm |
| bio 18 | Precipitation of Warmest Quarter | mm |
| bio 19 | Precipitation of Coldest Quarter | mm |
