# Supplementary material for: Exploring the Distribution of the Spreading Lethal Salamander Chytrid Fungus in Its Invasive Range in Europe – A Macroecological Approach
Source: PLoS One. 2016 Oct 31;11(10):e0165682. doi: 10.1371/journal.pone.0165682 (PMC5087956; doi:10.1371/journal.pone.0165682)
Supplement: S3 Table — (DOCX) [file pone.0165682.s008.docx]

**S3 Table. *Bsal* presences.**

| **Site name** | **Country** | **Longitude** | **Latitude** |
| --- | --- | --- | --- |
| Bunderbos* | Netherlands | 5.7473806 | 50.9131 |
| Putberg* | Netherlands | 5.9672583 | 50.855061 |
| Vijlenerbosch* | Netherlands | 5.93081 | 50.78258 |
| Meerssen | Netherlands | 5.7368056 | 50.908194 |
| Wormdal | Netherlands | 6.0789444 | 50.914422 |
| Pepinusbeekdal | Netherlands | 5.9235722 | 51.074517 |
| Eupen | Belgium | 6.0325861 | 50.608614 |
| Robertville | Belgium | 6.1182528 | 50.455664 |
| Liège | Belgium | 5.5794917 | 50.587575 |
| Weisse Wehe | Germany | 6.34569 | 50.71711 |
| Solchbachtal | Germany | 6.2701 | 50.70178 |
| Belgenbach | Germany | 6.2792 | 50.570533 |
| Roetgen# | Germany | 6.199235 | 50.678719 |
| Stolberg# | Germany | 6.292394 | 50.720145 |

Asterisks mark the first noted records (cf. Spitzen-van der Sluijs *et al.,* 2013) and hashtags unpublished records. Coordinates are in the WGS 1984 system.
