# Supplementary material for: Exploring the Distribution of the Spreading Lethal Salamander Chytrid Fungus in Its Invasive Range in Europe – A Macroecological Approach
Source: PLoS One. 2016 Oct 31;11(10):e0165682. doi: 10.1371/journal.pone.0165682 (PMC5087956; doi:10.1371/journal.pone.0165682)
Supplement: S4 Table — (DOCX) [file pone.0165682.s009.docx]

**S4 Tables**

**S4 Table A. Pearson correlation coefficients between variables of the ECA set for the different background extents.**

| **full** | **csu 5** | **csu 25** | **su 10-15** | **tr 10-15** | **cddn** | **cwdn** | **r 10** |
| --- | --- | --- | --- | --- | --- | --- | --- |
| **csu 5** | 1 | 0.352 | -0.216 | 0.447 | 0.065 | -0.261 | -0.346 |
| **csu 25** | 0.352 | 1 | -0.789 | 0.543 | 0.310 | -0.454 | -0.490 |
| **su 10-15** | -0.216 | -0.789 | 1 | -0.466 | -0.353 | 0.561 | 0.522 |
| **tr 10-15** | 0.447 | 0.543 | -0.466 | 1 | 0.278 | -0.549 | -0.529 |
| **cddn** | 0.065 | 0.310 | -0.353 | 0.278 | 1 | -0.371 | -0.348 |
| **cwdn** | -0.261 | -0.454 | 0.561 | -0.549 | -0.371 | 1 | 0.787 |
| **r 10** | -0.346 | -0.490 | 0.522 | -0.529 | -0.348 | 0.787 | 1 |
|  |  |  |  |  |  |  |  |
| **MCP 150** | **csu5** | **csu25** | **su10_15** | **tr10_15** | **cddn** | **cwdn** | **r10** |
| **csu5** | 1 | 0.298 | -0.170 | 0.509 | 0.045 | -0.323 | -0.370 |
| **csu25** | 0.298 | 1 | -0.786 | 0.471 | 0.271 | -0.431 | -0.429 |
| **su10_15** | -0.170 | -0.786 | 1 | -0.370 | -0.336 | 0.471 | 0.457 |
| **tr10_15** | 0.509 | 0.471 | -0.370 | 1 | 0.183 | -0.589 | -0.530 |
| **cddn** | 0.045 | 0.271 | -0.336 | 0.183 | 1 | -0.366 | -0.322 |
| **cwdn** | -0.323 | -0.431 | 0.471 | -0.589 | -0.366 | 1 | 0.799 |
| **r10** | -0.370 | -0.429 | 0.457 | -0.530 | -0.322 | 0.799 | 1 |
|  |  |  |  |  |  |  |  |
| **MCP 70** | **csu5** | **csu25** | **su10_15** | **tr10_15** | **cddn** | **cwdn** | **r10** |
| **csu5** | 1 | 0.483 | -0.301 | 0.721 | 0.053 | -0.477 | -0.476 |
| **csu25** | 0.483 | 1 | -0.687 | 0.584 | 0.137 | -0.548 | -0.511 |
| **su10_15** | -0.301 | -0.687 | 1 | -0.355 | -0.281 | 0.475 | 0.497 |
| **tr10_15** | 0.721 | 0.584 | -0.355 | 1 | 0.087 | -0.657 | -0.558 |
| **cddn** | 0.053 | 0.137 | -0.281 | 0.087 | 1 | -0.418 | -0.429 |
| **cwdn** | -0.477 | -0.548 | 0.475 | -0.657 | -0.418 | 1 | **0.820** |
| **r10** | -0.476 | -0.511 | 0.497 | -0.558 | -0.429 | **0.820** | 1 |

Absolute values > 0.8 are printed bold.

**S4 Table B. Pearson correlation coefficients between variables of the BIO set for the different background extents.**

| **full** | **bio 8** | **bio 10** | **bio 11** | **bio 15** | **bio 16** | **bio 17** |
| --- | --- | --- | --- | --- | --- | --- |
| **bio 8** | 1 | 0.302 | 0.086 | -0.447 | -0.498 | -0.213 |
| **bio 10** | 0.302 | 1 | 0.614 | 0.009 | -0.523 | -0.658 |
| **bio 11** | 0.086 | 0.614 | 1 | 0.006 | -0.319 | -0.459 |
| **bio 15** | -0.447 | 0.009 | 0.006 | 1 | 0.561 | -0.301 |
| **bio 16** | -0.498 | -0.523 | -0.319 | 0.561 | 1 | 0.541 |
| **bio 17** | -0.213 | -0.658 | -0.459 | -0.301 | 0.541 | 1 |
|  |  |  |  |  |  |  |
| **MCP 150** | **bio 8** | **bio 10** | **bio 11** | **bio 15** | **bio 16** | **bio 17** |
| **bio 8** | 1 | 0.331 | 0.174 | -0.474 | -0.522 | -0.250 |
| **bio 10** | 0.331 | 1 | 0.592 | 0.073 | -0.515 | -0.683 |
| **bio 11** | 0.174 | 0.592 | 1 | -0.076 | -0.350 | -0.449 |
| **bio 15** | -0.474 | 0.073 | -0.076 | 1 | 0.587 | -0.269 |
| **bio 16** | -0.522 | -0.515 | -0.350 | 0.587 | 1 | 0.564 |
| **bio 17** | -0.250 | -0.683 | -0.449 | -0.269 | 0.564 | 1 |
|  |  |  |  |  |  |  |
| **MCP 70** | **bio 8** | **bio 10** | **bio 11** | **bio 15** | **bio 16** | **bio 17** |
| **bio 8** | 1 | 0.506 | 0.365 | -0.612 | -0.522 | -0.351 |
| **bio 10** | 0.506 | 1 | 0.754 | -0.405 | -0.701 | -0.639 |
| **bio 11** | 0.365 | 0.754 | 1 | -0.275 | -0.521 | -0.597 |
| **bio 15** | -0.612 | -0.405 | -0.275 | 1 | 0.630 | 0.054 |
| **bio 16** | -0.522 | -0.701 | -0.521 | 0.630 | 1 | 0.753 |
| **bio 17** | -0.351 | -0.639 | -0.597 | 0.054 | 0.753 | 1 |

Absolute values > 0.8 are printed bold.

**S4 Table C. Pearson correlation coefficients between variables of the final ECA and BIO set.**

|  | **csu 5** | **csu 25** | **su 10-15** | **tr 10-15** | **cddn** | **cwdn** | **r 10** |
| --- | --- | --- | --- | --- | --- | --- | --- |
| **bio 8** | 0.118 | 0.182 | -0.346 | 0.247 | 0.239 | -0.596 | -0.454 |
| **bio 10** | 0.428 | **0.843** | **-0.823** | 0.756 | 0.345 | -0.617 | -0.583 |
| **bio 11** | 0.698 | 0.461 | -0.253 | 0.745 | 0.119 | -0.355 | -0.372 |
| **bio 15** | -0.084 | 0.202 | 0.005 | 0.014 | 0.090 | 0.381 | 0.277 |
| **bio 16** | -0.321 | -0.387 | 0.476 | -0.462 | -0.255 | **0.801** | **0.920** |
| **bio 17** | -0.330 | -0.654 | 0.554 | -0.633 | -0.408 | 0.586 | 0.751 |
|  |  |  |  |  |  |  |  |
| **MCP 150** | **csu 5** | **csu 25** | **su 10-15** | **tr 10-15** | **cddn** | **cwdn** | **r 10** |
| **bio 8** | 0.170 | 0.174 | -0.258 | 0.306 | 0.230 | -0.601 | -0.471 |
| **bio 10** | 0.434 | **0.874** | **-0.807** | 0.688 | 0.331 | -0.624 | -0.590 |
| **bio 11** | 0.750 | 0.410 | -0.203 | 0.746 | 0.089 | -0.417 | -0.386 |
| **bio 15** | -0.179 | 0.209 | -0.112 | -0.022 | 0.121 | 0.350 | 0.310 |
| **bio 16** | -0.348 | -0.337 | 0.392 | -0.486 | -0.230 | **0.805** | **0.922** |
| **bio 17** | -0.337 | -0.573 | 0.536 | -0.621 | -0.379 | 0.630 | 0.763 |
|  |  |  |  |  |  |  |  |
| **MCP 70** | **csu 5** | **csu 25** | **su 10-15** | **tr 10-15** | **cddn** | **cwdn** | **r 10** |
| **bio 8** | 0.291 | 0.407 | -0.286 | 0.451 | 0.291 | -0.645 | -0.445 |
| **bio 10** | 0.602 | **0.871** | -0.738 | 0.752 | 0.208 | -0.712 | -0.650 |
| **bio 11** | **0.802** | 0.601 | -0.338 | **0.903** | 0.008 | -0.574 | -0.494 |
| **bio 15** | -0.281 | -0.282 | 0.227 | -0.403 | -0.153 | 0.522 | 0.385 |
| **bio 16** | -0.492 | -0.531 | 0.514 | -0.626 | -0.415 | **0.851** | **0.928** |
| **bio 17** | -0.522 | -0.511 | 0.473 | -0.597 | -0.392 | 0.731 | **0.876** |

Absolute values > 0.8 are printed bold.

**S4 Table D. Pearson correlation coefficients between variables of the final ECA set (in row) and the remaining variables of the initial set (in column) for the full study area.**

| **full** | **csu 5** | **csu 25** | **su 10-15** | **tr 10-15** | **cddn** | **cwdn** | **r 10** |
| --- | --- | --- | --- | --- | --- | --- | --- |
| **su 5** | 0.702 | 0.640 | -0.473 | 0.765 | 0.238 | -0.484 | -0.509 |
| **su 10** | 0.626 | 0.767 | -0.663 | 0.764 | 0.327 | -0.617 | -0.635 |
| **su 15** | 0.534 | **0.839** | **-0.836** | 0.723 | 0.363 | -0.649 | -0.648 |
| **su 20** | 0.468 | **0.864** | **-0.858** | 0.675 | 0.345 | -0.628 | -0.620 |
| **su 25** | 0.441 | **0.875** | **-0.870** | 0.616 | 0.312 | -0.564 | -0.545 |
| **csu 10** | 0.594 | 0.666 | -0.598 | 0.623 | 0.317 | -0.510 | -0.581 |
| **csu 15** | 0.357 | 0.762 | -0.646 | 0.706 | 0.338 | -0.456 | -0.483 |
| **csu 20** | 0.369 | 0.696 | -0.729 | 0.573 | 0.312 | -0.521 | -0.518 |
| **tr 10** | 0.398 | 0.577 | -0.538 | **0.949** | 0.277 | -0.584 | -0.527 |
| **tr 15** | 0.310 | 0.553 | -0.556 | **0.805** | 0.249 | -0.561 | -0.473 |
| **tr 20** | 0.225 | 0.427 | -0.473 | 0.679 | 0.191 | -0.421 | -0.341 |
| **fd** | -0.605 | -0.238 | 0.017 | -0.741 | -0.049 | 0.219 | 0.251 |
| **cfd** | -0.414 | -0.425 | 0.282 | -0.568 | -0.209 | 0.342 | 0.394 |
| **id** | -0.613 | -0.593 | 0.449 | -0.781 | -0.302 | 0.576 | 0.614 |
| **r 1** | -0.379 | -0.688 | 0.657 | -0.716 | -0.498 | 0.758 | **0.811** |
| **r 20** | -0.320 | -0.377 | 0.401 | -0.373 | -0.226 | 0.609 | **0.857** |
| **cdd** | 0.144 | 0.504 | -0.417 | 0.482 | 0.091 | -0.310 | -0.330 |
| **cwd** | -0.277 | -0.326 | 0.400 | -0.499 | -0.176 | 0.677 | 0.615 |

Absolute values > 0.8 are printed bold.

**S4 Table E. Pearson correlation coefficients between variables of the final ECA set (in row) and the remaining variables of the initial set (in column) for the MCP 150 extent.**

| **MCP 150** | **csu 5** | **csu 25** | **su 10-15** | **tr 10-15** | **cddn** | **cwdn** | **r 10** |
| --- | --- | --- | --- | --- | --- | --- | --- |
| **su 5** | 0.729 | 0.589 | -0.434 | 0.754 | 0.220 | -0.544 | -0.525 |
| **su 10** | 0.650 | 0.728 | -0.625 | 0.725 | 0.296 | -0.662 | -0.656 |
| **su 15** | 0.545 | **0.815** | **-0.811** | 0.670 | 0.337 | -0.658 | -0.648 |
| **su 20** | 0.461 | **0.852** | **-0.840** | 0.600 | 0.310 | -0.616 | -0.603 |
| **su 25** | 0.420 | **0.894** | **-0.849** | 0.523 | 0.287 | -0.530 | -0.523 |
| **csu 10** | 0.572 | 0.621 | -0.584 | 0.631 | 0.333 | -0.543 | -0.568 |
| **csu 15** | 0.298 | 0.727 | -0.601 | 0.590 | 0.309 | -0.467 | -0.466 |
| **csu 20** | 0.306 | 0.691 | -0.744 | 0.462 | 0.335 | -0.523 | -0.546 |
| **tr 10** | 0.488 | 0.540 | -0.423 | **0.970** | 0.216 | -0.627 | -0.555 |
| **tr 15** | 0.404 | 0.582 | -0.454 | **0.820** | 0.240 | -0.615 | -0.530 |
| **tr 20** | 0.223 | 0.444 | -0.362 | 0.582 | 0.156 | -0.388 | -0.351 |
| **fd** | -0.670 | -0.201 | -0.014 | -0.770 | -0.027 | 0.304 | 0.270 |
| **cfd** | -0.402 | -0.380 | 0.235 | -0.563 | -0.209 | 0.349 | 0.349 |
| **id** | -0.634 | -0.521 | 0.378 | -0.771 | -0.287 | 0.601 | 0.589 |
| **r 1** | -0.389 | -0.621 | 0.588 | -0.658 | -0.490 | 0.769 | **0.825** |
| **r 20** | -0.303 | -0.306 | 0.358 | -0.380 | -0.215 | 0.657 | **0.861** |
| **cdd** | 0.107 | 0.405 | -0.289 | 0.358 | 0.041 | -0.232 | -0.266 |
| **cwd** | -0.298 | -0.241 | 0.262 | -0.485 | -0.103 | 0.634 | 0.562 |

Absolute values > 0.8 are printed bold.

**S4 Table F. Pearson correlation coefficients between variables of the final ECA set (in row) and the remaining variables of the initial set (in column) for the MCP 70 extent.**

| **MCP 70** | **csu 5** | **csu 25** | **su 10-15** | **tr 10-15** | **cddn** | **cwdn** | **r 10** |
| --- | --- | --- | --- | --- | --- | --- | --- |
| **su 5** | 0.142 | 0.680 | **0.806** | -0.671 | -0.608 | -0.506 | **0.872** |
| **su 10** | 0.239 | 0.766 | 0.738 | -0.741 | -0.687 | -0.629 | 0.796 |
| **su 15** | 0.270 | **0.805** | 0.674 | -0.725 | -0.689 | -0.785 | 0.735 |
| **su 20** | 0.228 | **0.840** | 0.587 | -0.669 | -0.640 | **-0.809** | 0.652 |
| **su 25** | 0.177 | **0.887** | 0.516 | -0.600 | -0.570 | **-0.806** | 0.585 |
| **csu 10** | 0.311 | 0.608 | 0.646 | -0.639 | -0.663 | -0.613 | 0.644 |
| **csu 15** | 0.160 | 0.712 | 0.580 | -0.701 | -0.687 | -0.606 | 0.724 |
| **csu 20** | 0.266 | 0.654 | 0.396 | -0.512 | -0.564 | -0.716 | 0.403 |
| **tr 10** | 0.093 | 0.625 | 0.704 | -0.688 | -0.573 | -0.371 | **0.987** |
| **tr 15** | 0.098 | 0.658 | 0.627 | -0.699 | -0.561 | -0.373 | **0.898** |
| **tr 20** | -0.104 | 0.243 | 0.405 | -0.279 | -0.153 | -0.056 | 0.620 |
| **fd** | 0.026 | -0.470 | -0.771 | 0.525 | 0.452 | 0.193 | **-0.931** |
| **cfd** | -0.026 | -0.395 | -0.525 | 0.406 | 0.343 | 0.184 | -0.651 |
| **id** | -0.202 | -0.604 | -0.739 | 0.697 | 0.631 | 0.407 | **-0.869** |
| **r 1** | -0.528 | -0.590 | -0.539 | **0.856** | **0.890** | 0.550 | -0.656 |
| **r 20** | -0.356 | -0.379 | -0.334 | 0.644 | **0.839** | 0.422 | -0.374 |
| **cdd** | 0.044 | 0.351 | 0.422 | -0.367 | -0.412 | -0.171 | 0.464 |
| **cwd** | -0.195 | -0.548 | -0.562 | 0.745 | 0.631 | 0.375 | -0.772 |

Absolute values > 0.8 are printed bold.

**S4 Table G. Pearson correlation coefficients between variables of the final BIO set (in row) and the remaining variables of the initial set (in column) for the full study area.**

| **full** | **bio 8** | **bio 10** | **bio 11** | **bio 15** | **bio 16** | **bio 17** |
| --- | --- | --- | --- | --- | --- | --- |
| **bio 1** | 0.236 | **0.940** | **0.832** | 0.019 | -0.473 | -0.637 |
| **bio 2** | 0.297 | 0.694 | 0.181 | -0.047 | -0.473 | -0.464 |
| **bio 3** | 0.312 | **0.271** | 0.092 | -0.237 | -0.398 | -0.165 |
| **bio 4** | 0.348 | **0.832** | 0.085 | -0.027 | -0.475 | -0.515 |
| **bio 5** | 0.219 | **0.968** | 0.570 | 0.106 | -0.456 | -0.654 |
| **bio 6** | 0.089 | 0.451 | 0.787 | 0.009 | -0.190 | -0.287 |
| **bio 7** | 0.188 | **0.801** | 0.197 | 0.109 | -0.389 | -0.551 |
| **bio 9** | -0.311 | 0.353 | 0.340 | 0.551 | 0.058 | -0.490 |
| **bio 12** | -0.421 | -0.655 | -0.444 | 0.214 | **0.902** | **0.809** |
| **bio 13** | -0.572 | -0.482 | -0.326 | 0.700 | **0.957** | 0.425 |
| **bio 14** | -0.045 | -0.616 | -0.407 | -0.360 | 0.385 | **0.854** |
| **bio 18** | 0.213 | -0.429 | -0.472 | -0.492 | 0.204 | 0.757 |
| **bio 19** | -0.578 | -0.456 | -0.165 | 0.578 | **0.909** | 0.480 |

Absolute values > 0.8 are printed bold.

**S4 Table H. Pearson correlation coefficients between variables of the final BIO set (in row) and the remaining variables of the initial set (in column) for the MCP 150 extent.**

| **MCP 150** | **bio 8** | **bio 10** | **bio 11** | **bio 15** | **bio 16** | **bio 17** |
| --- | --- | --- | --- | --- | --- | --- |
| **bio 1** | 0.294 | **0.907** | **0.861** | 0.010 | -0.483 | -0.644 |
| **bio 2** | 0.254 | 0.739 | 0.143 | 0.010 | -0.437 | -0.462 |
| **bio 3** | 0.319 | 0.544 | 0.275 | -0.227 | -0.440 | -0.284 |
| **bio 4** | 0.272 | 0.709 | -0.138 | 0.122 | -0.367 | -0.459 |
| **bio 5** | 0.226 | **0.967** | 0.509 | 0.168 | -0.428 | -0.650 |
| **bio 6** | 0.150 | 0.446 | 0.793 | -0.031 | -0.174 | -0.275 |
| **bio 7** | 0.131 | 0.690 | 0.000 | 0.191 | -0.321 | -0.480 |
| **bio 9** | -0.237 | 0.284 | 0.226 | 0.580 | 0.035 | -0.510 |
| **bio 12** | -0.465 | -0.635 | -0.444 | 0.255 | **0.905** | **0.812** |
| **bio 13** | -0.606 | -0.407 | -0.346 | 0.758 | **0.951** | 0.387 |
| **bio 14** | -0.079 | -0.638 | -0.356 | -0.330 | 0.426 | **0.852** |
| **bio 18** | 0.113 | -0.418 | -0.411 | -0.458 | 0.249 | 0.755 |
| **bio 19** | -0.582 | -0.384 | -0.166 | 0.600 | **0.903** | 0.459 |

Absolute values > 0.8 are printed bold.

**S4 Table I. Pearson correlation coefficients between variables of the final BIO set (in row) and the remaining variables of the initial set (in column) for the MCP 70 extent.**

| **MCP 70** | **bio 8** | **bio 10** | **bio 11** | **bio 15** | **bio 16** | **bio 17** |
| --- | --- | --- | --- | --- | --- | --- |
| **bio 1** | 0.490 | **0.930** | **0.932** | -0.398 | -0.664 | -0.656 |
| **bio 2** | 0.239 | 0.606 | 0.078 | -0.170 | -0.420 | -0.332 |
| **bio 3** | 0.269 | 0.552 | 0.268 | -0.184 | -0.364 | -0.306 |
| **bio 4** | 0.273 | 0.544 | -0.133 | -0.227 | -0.398 | -0.223 |
| **bio 5** | 0.399 | **0.960** | 0.617 | -0.295 | -0.629 | -0.598 |
| **bio 6** | 0.299 | 0.569 | **0.848** | -0.219 | -0.325 | -0.393 |
| **bio 7** | 0.149 | 0.483 | -0.088 | -0.112 | -0.356 | -0.269 |
| **bio 9** | -0.239 | 0.048 | 0.094 | 0.486 | -0.027 | -0.407 |
| **bio 12** | -0.471 | -0.691 | -0.536 | 0.377 | **0.938** | **0.906** |
| **bio 13** | -0.626 | -0.714 | -0.593 | 0.734 | **0.967** | 0.692 |
| **bio 14** | -0.291 | -0.671 | -0.616 | 0.166 | 0.770 | **0.881** |
| **bio 18** | 0.055 | -0.339 | -0.342 | -0.327 | 0.448 | **0.817** |
| **bio 19** | -0.593 | -0.565 | -0.349 | 0.634 | **0.887** | 0.637 |

Absolute values > 0.8 are printed bold.
